# Acetylation of MAT IIα represses tumour cell growth and is decreased in human hepatocellular cancer

**DOI:** 10.1038/ncomms7973

**Published:** 2015-04-30

**Authors:** Hong-Bin Yang, Ying-Ying Xu, Xiang-Ning Zhao, Shao-Wu Zou, Ye Zhang, Min Zhang, Jin-Tao Li, Feng Ren, Li-Ying Wang, Qun-Ying Lei

**Affiliations:** 1 Key Laboratory of Metabolism and Molecular Medicine, Ministry of Education, and Department of Biochemistry and Molecular Biology, School of Basic Medical Sciences, Fudan University, Shanghai 200032, China; 2 Department of Hepatopancreatobiliary Surgery, Shanghai 10th People's Hospital, Tong Ji University, Shanghai 200072, China; 3 Cancer Metabolism Lab, Institutes of Biomedical Sciences, Fudan University, Shanghai 200032, China; 4 Collaborative Innovation Center of Systems Biomedicine, Shanghai Jiaotong University, Shanghai 200025, China

## Abstract

Metabolic alteration is a hallmark of cancer. Dysregulation of methionine metabolism is implicated in human liver cancer. Methionine adenosyltransferase IIα (MAT IIα) is a key enzyme in the methionine cycle, catalysing the production of *S*-adenosylmethionine (SAM), a key methyl donor in cellular processes, and is associated with uncontrolled cell proliferation in cancer. Here we show that P300 acetylates MAT IIα at lysine residue 81 and destabilizes MAT IIα by promoting its ubiquitylation and subsequent proteasomal degradation. Conversely, histone deacetylase-3 deacetylates and stabilizes MAT IIα by preventing its proteasomal degradation. Folate deprivation upregulates K81 acetylation and destabilizes MAT IIα to moderate cell proliferation, whereas a single mutation at K81 reverses the proliferative disadvantage of cancer cells upon folate deprivation. Moreover, MAT IIα K81 acetylation is decreased in human hepatocellular cancer. Collectively, our study reveals a novel mechanism of MAT IIα regulation by acetylation and ubiquitylation, and a direct functional link of this regulation to cancer development.

## Abstract (web summary)

Folate plays an essential role in dividing cells and is regulated by methionine adenosyltransferase (MAT), where a switch from MAT Iα to MAT IIα expression seems to promote liver cancer progression. Here the authors demonstrate that MAT IIα stability is regulated by acetylation and this regulation is important for tumour growth.

## Graphical abstract

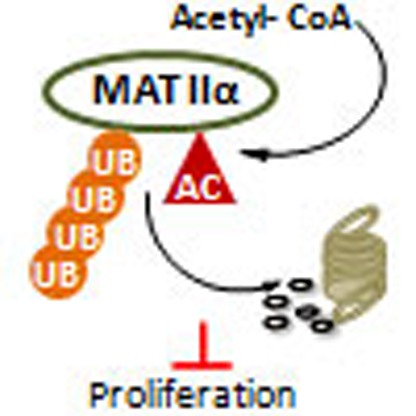

Folate is essential for rapidly proliferating cells and plays a pivotal role in one-carbon metabolism. Its metabolic derivative tetrahydrofolate (FH4), known as the carrier of one-carbon units, is involved in *de novo* synthesis of thymidylate and purine, amino-acid inter-conversion and so on^1^. Coupling with methionine cycle, which produces the major methyl donor *S*-adenosylmethionine (SAM), folate metabolism also presents methyl-groups to global methylation of macromolecules.

Methionine adenosyltransferase (MAT) is the key enzyme in methionine cycle, catalysing biosynthesis of SAM. In mammals, two distinct genes, *MAT1A* and *MAT2A*, encode two homologous catalytic subunits—α1 and α2, respectively^2^. *MAT1A* mainly expresses in healthy hepatocytes. Its encoding product, the α1 subunits, assemble into two MAT isozymes, MAT III (dimer) and MAT I (tetramer)^2^. In contrast, *MAT2A* and its coding product, MAT IIα (dimer formed from α2 subunits), is widely expressed in all exhepatic tissues at relatively low level under normal conditions^3^, whereas overexpressed in various human epithelial tumours, including gastric, colon and liver cancer^4^,^5^,^6^,^7^.

Studies indicated that enhanced *MAT2A* expression boosts cancer cell proliferation and potentially promotes tumour development and progression^2^,^8^,^9^. In addition, *MAT2A*, but not *MAT1A*, is found expressed in fetal liver, whereas healthy adult liver expresses mainly *MAT1A*^3^. MAT expression is progressively switched from *MAT2A* to *MAT1A* during liver development, whereas is reversed to *MAT2A* again during liver malignant transformation. In addition, knockdown *MAT2A* dramatically suppresses tumour cell proliferation and induces cell cycle arrest and apoptosis^7^,^10^,^11^. Therefore, the transcriptional switch from *MAT1A* to *MAT2A* is believed to play a role in facilitating cancer cell survival and proliferation^11^,^12^.

*MAT2A* expression is regulated at both transcriptional and post-transcriptional levels. Sp1 (transcription factor: specificity protein 1), c-Myb (transcription activator: avian myeloblastosis viral oncogene), NF-κB (nuclear factor of kappa, enhancer in B cells) and AP-1 (adaptor-related protein complex 1) were identified as the *trans*-activating factors involved in *MAT2A* transcriptional upregulation^13^,^14^. Tumour-necrosis factor-α upregulates *MAT2A* via NF-κB and AP-1 (ref. 14). Moreover, histone acetylation, promoter methylation and mRNA stabilization were also reported to regulate *MAT2A* expression^15^. Although regulation of *MAT2A* expression was heavily studied at transcriptional and post-transcriptional levels, its post-translational regulation remains largely unknown.

Covalent lysine acetylation has been identified as an evolutionarily conserved modification in metabolic enzymes, and plays critical roles in regulation of multiple enzymes^16^,^17^,^18^. In this study, we demonstrate that MAT IIα protein is acetylated at lysine residue 81 in response to folate deprivation. Acetylation promotes MAT IIα ubiquitylation and its subsequent proteasomal degradation, inhibiting tumour cell proliferation. Our observations reveal a novel mechanism of MAT IIα upregulation in human cancers.

## Results

### MAT IIα is acetylated at lysine 81

**Figure 1:**
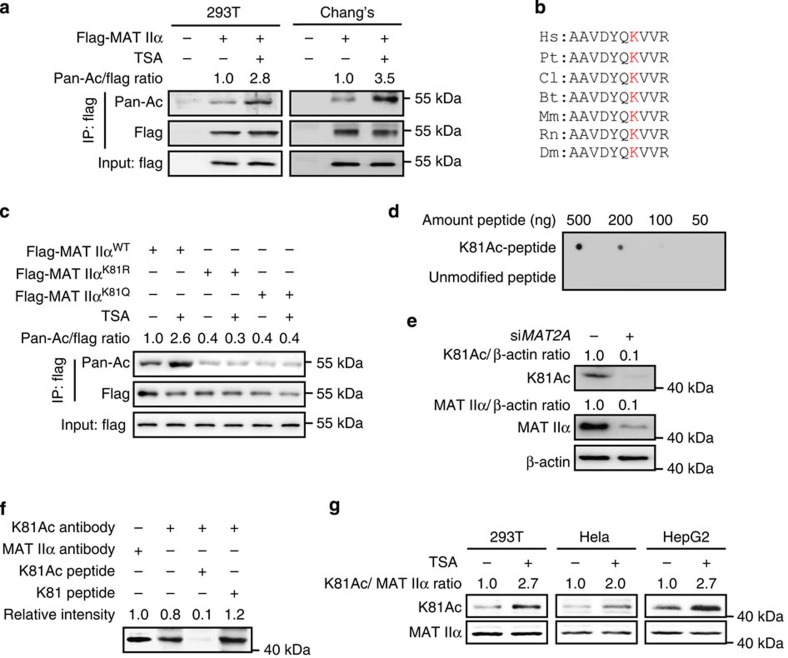
MAT IIα is acetylated at lysine 81. (**a**) Exogenous MAT IIα is acetylated. Flag-MAT IIα was transfected into HEK293T and Chang's cells followed by TSA treatment (10 μM) for 18 h. Flag-MAT IIα acetylation was detected by anti-acetyllysine (Pan-Ac) antibody. (**b**) Alignment of MAT IIα lysine 81 and adjacent protein sequence from different organisms. Hs: *Homo Sapient* (human); Pt: *Pan troglodytes* (chimpanzee); Cl: *Canis lupus familiaris* (dog); Bt: *Bos taurus* (bull); Mm: *Mus musculus* (mouse); Rn: *Rattus norvegicus* (Norway rat); Dm: *Drosophila melanogaster* (fruit fly). (**c**) TSA increases acetylation level of wild-type MAT IIα but not mutants. Ectopically expressed wild-type MAT IIα, K81R and K81Q were transfected into HEK293T cells followed by TSA treatment, MAT IIα acetylation was analysed by western blotting. (**d**) Specificity of K81 site-specific acetylation antibody was determined by dot blotting assay. (**e**) *MAT2A* knockdown confirmed the K81Ac antibody detection. HEK293T cells were transfected with *MAT2A* si*RNA* or scramble control and cell lysates were subjected to western blotting. Signal detected by K81Ac antibody decreased as MAT IIα was knocked down. (**f**) K81Ac antibody is specific to MAT IIα K81 acetylation. HEK293T cells were lysed and directly subjected to western blotting. The four repetitively loaded lanes were clipped into four membranes and were exposed to antibodies as indicated, separately. For peptide block, acetylated K81 peptide (the antigen we used for K81Ac antibody generation) or non-acetylated K81 peptide (mimicking MAT IIα protein) were added to the diluted K81Ac antibodies (1:200) in a final concentration of 300 μg ml^-1^, cultivated at 37 °C for 1 h before applying to membranes. Four clipped membranes were aligned and exposed together when developing film to make sure their relative signal intensities are comparable. (**g**) TSA promotes MAT IIα acetylation at K81 in different cell lines. HEK293T, Hela and HepG2 cells were treated as indicated. K81 acetylation was detected by K81Ac antibody and normalized against MAT IIα protein.

Previous mass spectrometry (MS) analyses indicated that MAT IIα was potentially an acetylated protein (Supplementary Fig. 1a). To confirm the acetylation modification, pan acetyl-lysine antibody was used to detect the acetylation level of ectopically expressed MAT IIα. Result showed that MAT IIα was indeed acetylated in HEK293T and Chang's cells (Fig. 1a). Furthermore, trichostatin A (TSA), an inhibitor of histone deacetylase HDAC family I, II and IV, increased the acetylation level of flag-MAT IIα approximately two- to threefold (Fig. 1a). Given that lysine (K) 81 is the only putative acetylation site identified in MAT IIα by MS (Fig. 1b and Supplementary Fig. 1a), and is evolutionarily conserved from *D. melanogaster* to mammals, we mutated K81 to arginine (R) and glutamine (Q) and found that both mutations resulted in a significant reduction in acetylation (Fig. 1c). Notably, TSA treatment dramatically increased the acetylation level of wild-type MAT IIα, but not K81R or K81Q mutant, indicating K81 might be the primary acetylation site of MAT IIα (Fig. 1c). To further confirm acetylation of K81, we generated a K81 site-specific antibody specifically targeting to acetylated K81 residue in MAT IIα (designated as ‘K81Ac antibody' henceforth). Dot blotting assay was performed to characterize the specificity of this antibody and found that K81Ac antibody preferentially detected K81 acetylated, but not unmodified peptide (Fig. 1d). In addition, strong and specific signal of K81 acetylation was observed in ectopically expressed wild-type MAT IIα but not K81R mutant (Supplementary Fig. 1b). Further results showed that both *MAT2A* knockdown (Fig. 1e) and peptide blocking by K81 acetylated peptide (Fig. 1f) significantly reduced K81Ac signal, indicating a high specificity of this antibody to MAT IIα K81 acetylation. More importantly, with the help of this site-specific antibody, we detected enhanced K81 acetylation signal of endogenous MAT IIα in different cell lines upon TSA treatment (Fig. 1g). These results indicate that K81 is the major acetylation site of MAT IIα under the tested condition.

### Folate decreases K81 acetylation and stabilizes MAT IIα

**Figure 2:**
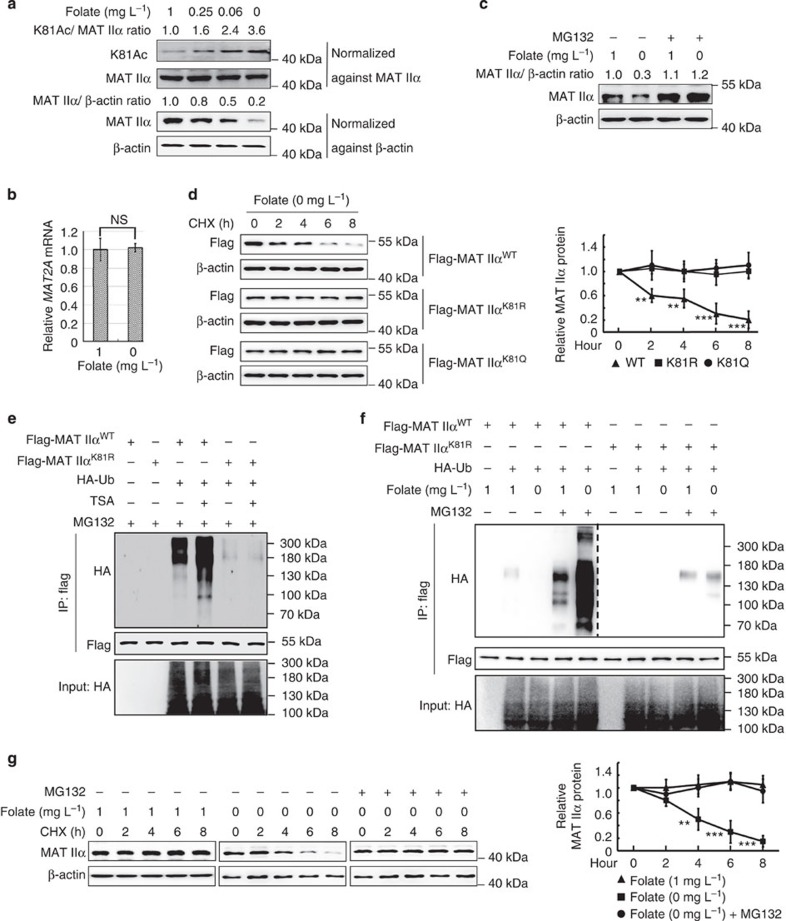
Folate decreases K81 acetylation and stabilizes MAT IIα. (**a**) Folate deprivation increases K81 acetylation of MAT IIα and decreases its protein level. HEK293T cells were cultured in medium containing folate of different concentrations as indicated for 48 h. Western blotting was used to determine MAT IIα protein and K81 acetylation levels. (**b**) Folate-deprivation has no effect on *MAT2A* mRNA level. *MAT2A* mRNA was determined by qPCR and normalized against β-actin. Error bars represent ±s.d. of triplicate experiments. The two-tailed Student's *t*-test was used. NS denotes no significance. (**c**) MG132 rescues the decrease in MAT IIα protein induced by folate deprivation. HEK293T cells were cultured as indicated and treated with either dimethylsulphoxide (DMSO; solvent) or 10 μM MG132 for 6 h. Cell lysates were analysed by western blotting. (**d**) Folate deprivation exclusively shortens the half-life of wild-type MAT IIα, but not K81R or K81Q mutant. HEK293 stable cell lines expressing wild-type MAT IIα, or K81R/K81Q mutants were cultured in folate-deprived condition for 48 h, CHX (10 μg ml^-1^) treatment was applied for different time course before harvest. MAT IIα protein levels were determined by western blotting (left panel). The right panel showcases relative protein amounts of different groups. Error bars represent ±s.d. of triplicate experiments. The two-tailed Student's *t*-test was used. **P<0.01; ***P<0.001. (**e**) K81R mutation blocks MAT IIα ubiquitylation induced by TSA. HEK293T cells were transfected with the indicated plasmids. After TSA and MG132 treatment as indicated, ubiquitylation of purified flag-MAT IIα proteins were determined. (**f**) Folate deprivation promotes ubiquitylation of wild-type MAT IIα but not K81R mutant. HEK293T cells were transfected as indicated. Ubiquitylation of flag-MAT IIα protein was determined by western blotting. (**g**) MG132 stabilizes MAT IIα upon folate deprivation. HEK293T cells were cultured under normal or folate-deprived condition, followed by CHX and MG132 treatment. Cell lysates were directly subjected to western blotting (left panel). The right panel showcases relative protein amounts of different groups. Error bars represent ±s.d. of triplicate experiments. The two-tailed Student's *t*-test was used. **P<0.01; ***P<0.001.

Folate's metabolic derivative tetrahydrofolate plays an essential role in transferring one-carbon units. Restoration of methionine from homocysteine in methionine cycle greatly depends on methyl-groups (one type of one-carbon units) presented from folate metabolism. Therefore, methionine cycle is tightly related to folate availability and one-carbon unit metabolism. As serine hydroxymethyl transferation and glycine cleavage are two major sources of one-carbon units, methionine cycle is also related to serine and glycine metabolisms. To determine whether K81 acetylation of MAT IIα is dynamically regulated by serine, glycine or folate *in vivo*, we cultured HEK293T cells in medium containing these metabolites with different concentrations and found that K81 acetylation level of MAT IIα increased as concentrations of folate decreased (Fig. 2a, upper panel). In contrast to this, serine and glycine showed no effect on MAT IIα K81 acetylation (data not shown). More interestingly, steady-state level of MAT IIα protein decreased as concentrations of folate decreased (Fig. 2a, lower panel). Similar results were obtained in human hepatocellular carcinoma cell line—Huh7 (Supplementary Fig. 2a). These results indicate a potential inverse correlation between MAT IIα acetylation and protein levels in response to folate concentration.

### K81 acetylation promotes MAT IIα degradation

To determine whether downregulation of MAT IIα protein by folate occurs at transcriptional level, quantitative reverse transcription–PCR was performed. Results showed that folate treatment had no effect on MAT IIα mRNA level (Fig. 2b), indicating that folate regulates MAT IIα protein mainly at the post-transcriptional level. To determine whether acetylation induced MAT IIα degradation is mediated by proteasomal pathway, we treated HEK293T cells with proteasome inhibitor MG132 and found both TSA and folate deprivation induced decrease in MAT IIα protein can be readily restored by MG132 (Fig. 2c and Supplementary Fig. 2b), suggesting K81 acetylation in response to TSA or folate deprivation might target MAT IIα protein to proteasomal degradation. The fact that MAT IIα was accumulated in HEK293T, H1299 and U937 cells after treated with MG132 further supported this speculation (Supplementary Fig. 2c). In addition, TSA treatment combined with protein synthesis inhibitor cycloheximide (CHX) dramatically decreased the half-life of endogenous MAT IIα (Supplementary Fig. 2d). These results indicate that TSA- and folate deprivation-induced MAT IIα protein degradation is proteasomal dependent. To further test the possibility that TSA or folate deprivation promotes MAT IIα degradation via K81 acetylation, half-lives of wild-type, K81R and K81Q MAT IIα were determined by CHX chase experiment. Wild-type MAT IIα, but not the mutants, was found to display a shortened half-life under folate-deprived condition (Fig. 2d), indicating proteasomal degradation of MAT IIα might be related to K81 acetylation. In addition, we employed ubiquitylation ladder assay and observed a significant increase in wild-type MAT IIα ubiquitylation in the presence of TSA or upon folate deprivation, whereas ubiquitylation of K81R mutation was mostly blocked and no longer respond to either TSA or folate deprivation (Fig. 2e,f). Consistently, we found MG132 could block TSA- or folate deprivation-induced MAT IIα destabilization (Fig. 2g and Supplementary Fig. 2e). Collectively, these results demonstrate that K81 acetylation of MAT IIα promotes its degradation via ubiquitylation-mediated proteasomal degradation.

### UBR4 targets MAT IIα for degradation

**Figure 3:**
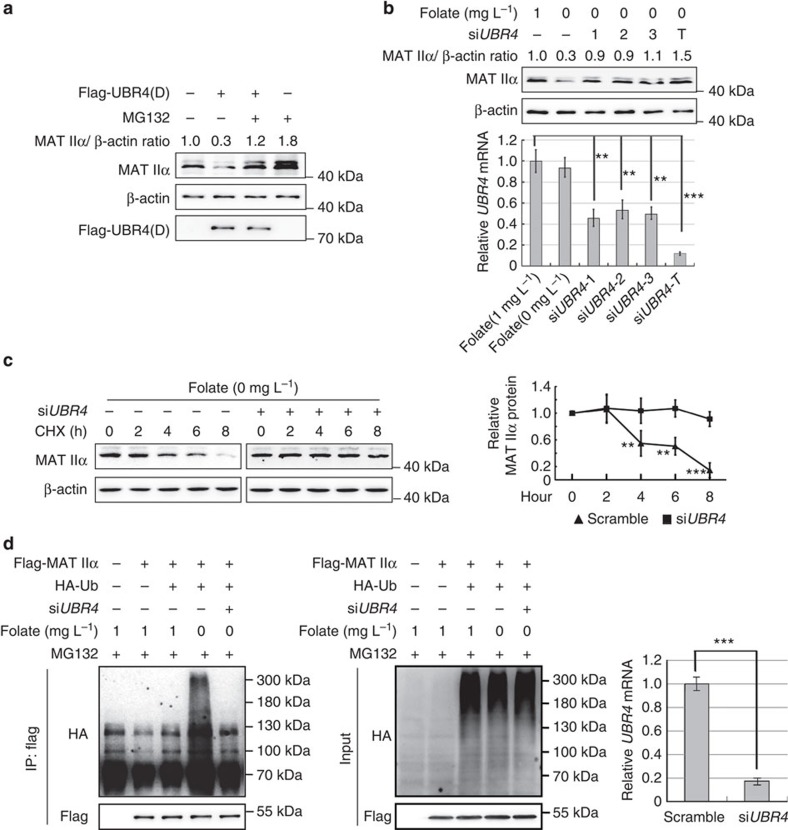
UBR4 targets MAT IIα for degradation. (**a**) UBR4 overexpression decreases MAT IIα protein. HEK293T cells were transfected as indicated (UBR4 (D) represents a truncated form of UBR4 protein, with substrate-binding and catalytic domains only), followed by MG132 treatment. Cell lysates were directly subjected to western blotting. (**b**) *UBR4* knockdown rescues MAT IIα protein reduced by folate deprivation. Three different si*RNA* oligos si*UBR4*-1, -2 and -3 were transfected, respectively, or together (si*UBR4*-T) into HEK293T cells. Protein levels of MAT IIα were determined by western blotting. *UBR4* knockdown efficiency was analysed by qPCR. Values were normalized against β-actin and compared with the relative mRNA of folate (1 mg l^-1^) group (set as 1.0). Error bars represent ±s.d. of triplicate experiments. The two-tailed Student's *t*-test was used. **P<0.01; ***P<0.001. (**c**) *UBR4* knockdown increases MAT IIα stability. HEK293T cells were transfected with si*UBR4* or control. CHX chase experiment was performed and MAT IIα protein was determined by western blotting (left panel). The right panel showcases relative protein amounts of different groups. Error bars represent ±s.d. of triplicate experiments. The two-tailed Student's *t*-test was used. **P<0.01; ***P<0.001. (**d**) *UBR4* knockdown blocks folate-deprivation induced-ubiquitylation of MAT IIα. HEK293T cells were transfected as indicated and cultured in folate containing or deprived culture medium. Ubiquitylation assay was conducted. The efficiency of *UBR4* knockdown was validated by qPCR. Error bars represent ±s.d. of triplicate experiments. The two-tailed Student's *t*-test was used. ***P<0.001.

Three types of enzymes are required in the process of ubiquitylation: ubiquitin-activating enzymes (E1s), ubiquitin-conjugating enzymes (E2s) and ubiquitin-ligases (E3s), among which, only E3s define the target specificity of ubiquitylation reaction. To further investigate the mechanism of TSA- or folate deprivation-induced MAT IIα proteasomal degradation, we established an HEK293-derived cell line stably expressing both streptavidin-binding protein and flag-tagged MAT IIα, cultured upon folate deprivation and purified MAT IIα protein using tandem affinity purification to identify its E3 ligase via MS. Among the consistently identified interacting proteins, we identified UBR4 (ubiquitin protein ligase E3 component n-recognin 4, belongs to the zinc finger E3 ligase family) as a putative E3 ligase for MAT IIα. To verify whether UBR4 is an E3 ligase for MAT IIα, we overexpressed *UBR4* (D) (expressing a truncated form of UBR4 protein, with substrate-binding and catalytic domains only) in HEK293T cells and found a significant drop in MAT IIα protein, which was totally reversed by adding MG132 to the culture medium (Fig. 3a). Furthermore, knocking down *UBR4* expression by three independent short interfering RNAs (siRNAs) could instantly block folate deprivation-induced MAT IIα degradation (Fig. 3b). Consistently, CHX chase experiment showed that *UBR4* knockdown could totally block the decreased half-life of MAT IIα upon folate deprivation (Fig. 3c). Moreover, *UBR4* knockdown increases MAT IIα protein level and its acetylation level (Supplementary Fig. 3, left panel). The efficacy of *UBR4* knockdown was validated by quantitative PCR (qPCR; Supplementary Fig. 3, right panel). In addition, we performed ubiquitin ladder experiment to visualize ubiquitylation. Consistent with previous findings, polyubiquitylation signal of MAT IIα was detected upon folate deprivation (Fig. 3d), whereas HA-tagged ubiquitin could no longer be detected at high molecular weight after knocking down *UBR4* expression (for quantification of *UBR4* mRNA level, please refer to Fig. 3d). Collectively, these results verify UBR4 as an E3 ligase participating in polyubiquitylation-mediated proteasomal degradation of MAT IIα.

### P300 acetylates MAT IIα at K81

**Figure 4:**
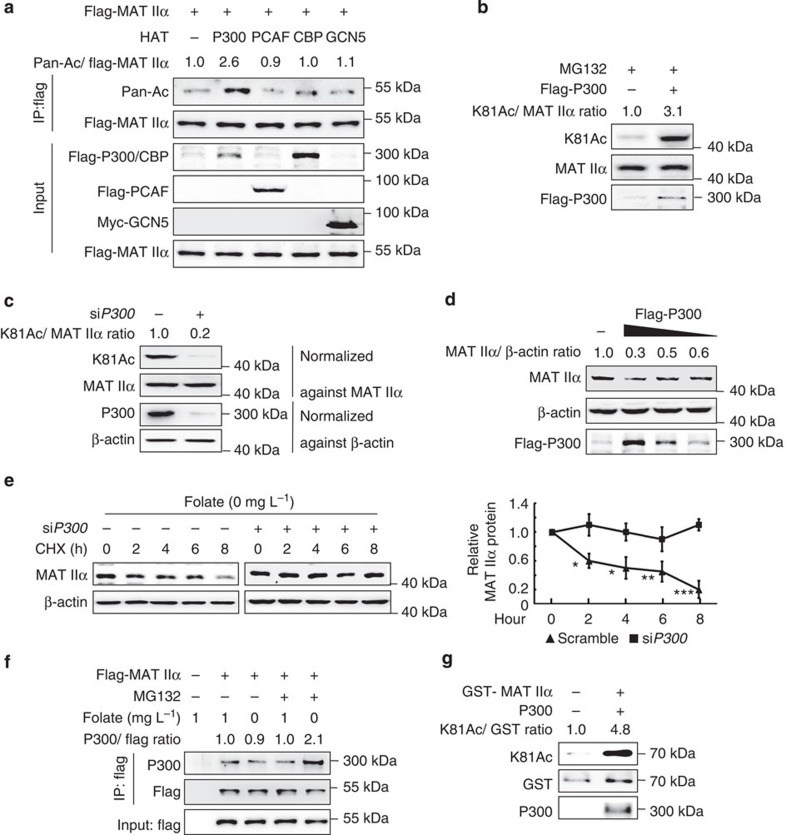
P300 acetylates MAT IIα. (**a**) Overexpression of P300, not other HATs, increases acetylation of exogenous MAT IIα. HEK293T cells were transfected as indicated and acetylation of flag-MAT IIα was determined using Pan-Ac antibody. (**b**) Overexpression of P300 increases K81 acetylation of endogenous MAT IIα. HEK293T cells were transfected with flag-P300 and K81 acetylation of MAT IIα was determined using K81Ac antibody. (**c**) *P300* knockdown decreases MAT IIα K81 acetylation. HEK293T cells were transfected with si*P300* or control and MAT IIα acetylation levels were determined by western blotting. (**d**) P300 decreases protein levels of MAT IIα. Flag-P300 was transfected in declined doses into HEK293T cells and cell lysates were measured by western blotting. (**e**) *P300* knockdown increases MAT IIα stability. HEK293T cells were transfected with si*P300* or control and cultured under folate-deprived condition before CHX was added and treated for indicated durations. Levels of endogenous MAT IIα protein were determined by western blotting and normalized against β-actin (left panel). The right panel showcases relative protein amounts of different groups. Error bars represent ±s.d. of triplicate experiments. The two-tailed Student's *t*-test was used. *P<0.05; **P<0.01; ***P<0.001. (**f**) Folate-deprivation enhances MAT IIα–P300 interaction. HEK293T cells were transfected with flag-tagged MAT IIα and cultured in normal or folate-deprived conditions. The interaction between flag-MAT IIα and endogenous P300 was determined by co-IP followed by western blotting. (**g**) P300 acetylates MAT IIα at K81 *in vitro*. Ectopically expressed GST-MAT IIα from BL21 *E. coli* was purified through GST antibody-conjugated beads and incubated with P300 (immunoprecipitation-purified and eluted). K81 acetylation levels were analysed using K81Ac antibody and normalized against GST-MAT IIα.

**Figure 5:**
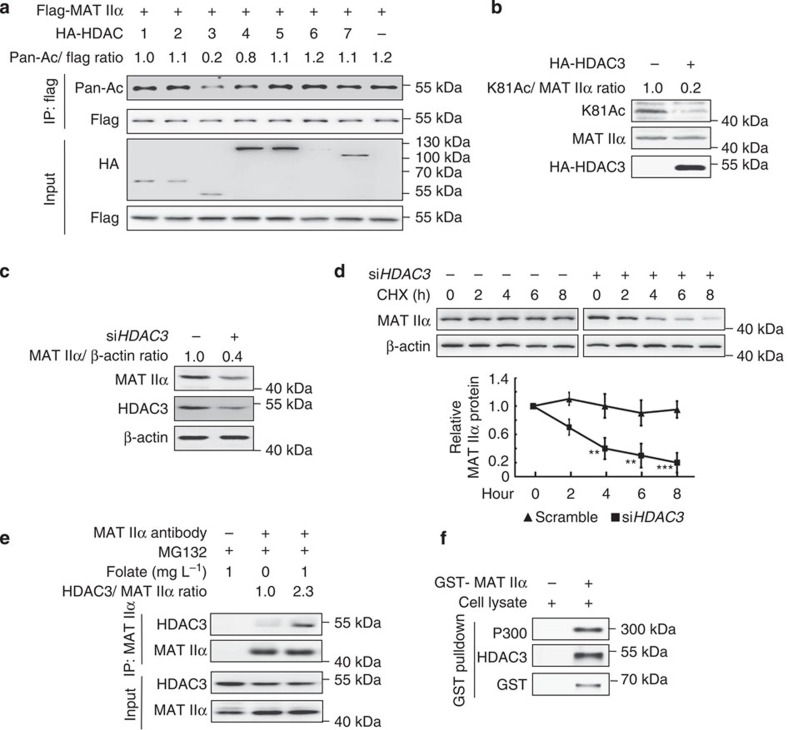
HDAC3 deacetylates MAT IIα. (**a**) Overexpression of HDAC3, but not other HDACs, decreases the acetylation level of MAT IIα. Each of the HA-tagged HDAC1-7 was co-transfected with flag-tagged MAT IIα into HEK293T cells and the acetylation levels of MAT IIα were determined by standard western blotting. (**b**) HDAC3 decreases K81 acetylation of endogenous MAT IIα. HEK293T cells were transfected with HA-HDAC3. MAT IIα K81 acetylation was detected using K81Ac antibody. (**c**) *HDAC3* knockdown decreases MAT IIα protein. HEK293T cells were transfected with si*HDAC3* or control. Cells were harvested 48 h after transfection, and lysates were analysed by western blotting. (**d**) *HDAC3* knockdown destabilizes MAT IIα. HEK293T cells were transfected with si*HDAC3* or control, respectively. CHX chase treatment was applied as indicated. Cell lysates were directly subjected to western blotting (upper panel) and normalized against β-actin. The lower panel showcases relative protein amounts of different groups. Error bars represent ±s.d. of triplicate experiments. The two-tailed Student's *t*-test was used. **P<0.01; ***P<0.001. (**e**) Folate increases the interaction between HDAC3 and MAT IIα. HEK293T cells were cultured with or without folate for 48 h and treated with MG132 for 6 h before harvest. Interaction between endogenous MAT IIα and HDAC3 was determined by co-IP and western blotting. (**f**) GST-MAT IIα can readily pull-down P300 and HDAC3. BL21 *E. coli* transformed with pGEX**-GST-MAT IIα plasmid was induced (or not induced) by isopropyl-β-D-thiogalactoside. Protein was then purified through GST antibody-conjugated column and incubated with HEK293T cell lysates (lysed by 0.3% Nonidet P40 buffer) before re-purified through immunoprecipitation and subjected to western blotting.

To identify the acetyl-transferase responsible for MAT IIα K81 acetylation, we co-transfected fourHATs (histone acetyl-transferases), *P300* (E1A binding protein), *PCAF* (P300/CBP-associated factor, also known as K (lysine) acetyl-transferase 2B, *KAT2B*), *CBP* (CREB-binding protein) and *GCN5* (*KAT2A*), individually with *MAT2A* into HEK293T cells, and found P300 overexpression increased MAT IIα acetylation, whereas others did not (Fig. 4a). In addition, overexpression of P300 increased endogenous K81 acetylation of MAT IIα (Fig. 4b). To further verify whether P300 acetylates MAT IIα at K81, we designed experiment shown in Supplementary Fig. 4, and proved that P300 overexpression only increases acetylation of wild-type MAT IIα, but not the K81R/Q mutant. Conversely, knocking down *P300* by RNA interference significantly reduced K81 acetylation of endogenous MAT IIα (Fig. 4c). P300 decreased MAT IIα protein level in a dose-dependent manner (Fig. 4d), whereas *P300* knockdown rescued folate deprivation-induced MAT IIα destabilization (Fig. 4e). Moreover, we found folate-deprivation increased the interaction between endogenous MAT IIα and P300 (Fig. 4f), and this interaction was further verified by glutathione *S*-transferase (GST) pull-down (Fig. 5f). To further examine the hypothesis that P300 is the acetyl-transferase of MAT IIα, *in vitro* acetylation assay was conducted. As shown in Fig. 4g, recombinant P300 instantly acetylated MAT IIα at K81. Collectively, our results demonstrate that P300 is the acetyl-transferase responsible for MAT IIα acetylation at K81.

### HDAC3 deacetylates MAT IIα at K81

According to the previous results that TSA, an inhibitor of type I, II and IV HDACs, increased K81 acetylation of MAT IIα, we postulated that HDACs might be involved in MAT IIα deacetylation. We co-expressed HDAC1-7 individually with MAT IIα, and found that overexpression of HDAC3, but not other tested HDACs, decreased the acetylation level of ectopically expressed MAT IIα (Fig. 5a and Supplementary Fig. 5). Thereafter, we overexpressed HDAC3 in HEK293T cells, and found a decrease in K81 acetylation of endogenous MAT IIα (Fig. 5b). Notably, *HDAC3* knockdown decreased the steady protein level of MAT IIα (Fig. 5c). Furthermore, we found *HDAC3* knockdown led to the destabilization of MAT IIα (Fig. 5d), further supported that deacetylation stabilizes MAT IIα. Previous study by Katoh *et al*. identified HDAC3 as a putative MAT IIα-interacting protein by MS analysis^19^. To verify this interaction between HDAC3 and MAT IIα, we performed co-immunoprecipitation (co-IP) experiment and found that interaction indeed exists between these two proteins, and the interaction could be further enhanced in the presence of folate (Fig. 5e). Similarly, GST pull-down was performed to further verify this interaction (Fig. 5f). Taken together, HDAC3 is the deacetylase for MAT IIα K81 acetylation.

### K81 mutants promote tumour growth *in vitro* and *in vivo*

**Figure 6:**
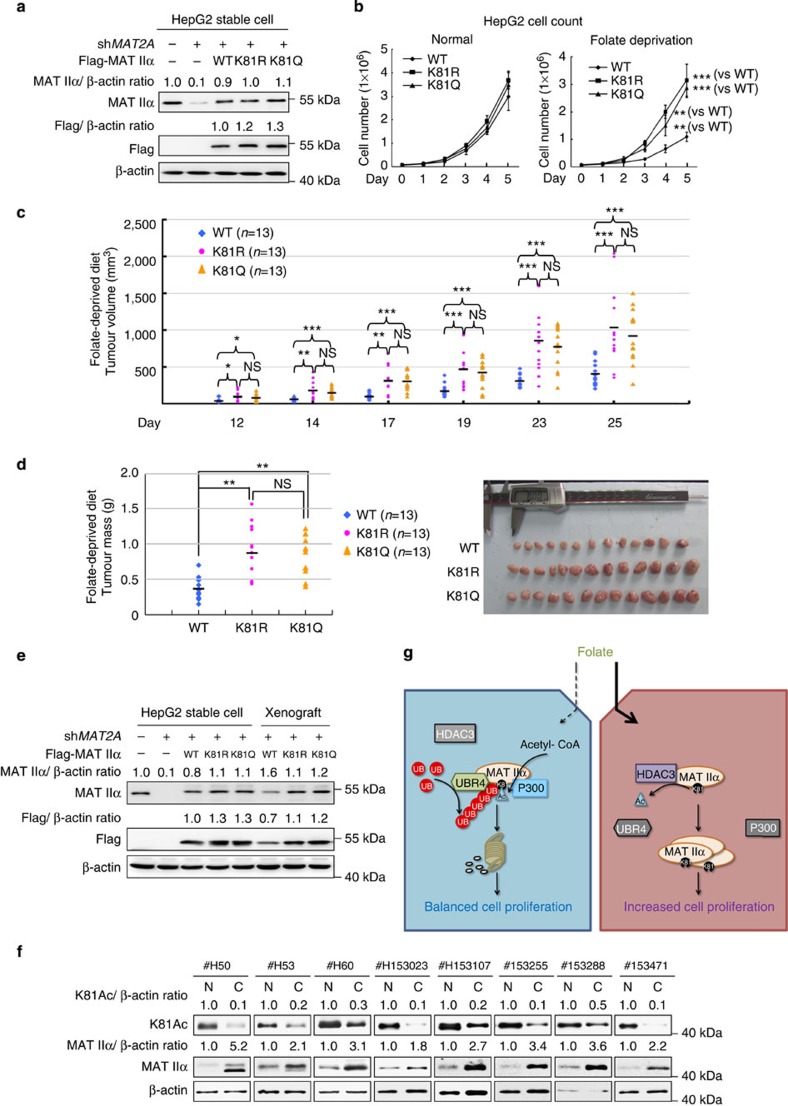
K81 mutations promote tumour cell growth *in vitro* and *in vivo.* (**a**) Verification of HepG2 stable cell lines. Knockdown efficiency and re-expression levels of wild-type or K81R/Q mutants were determined by western blotting. (**b**) K81R and K81Q mutations reverse the proliferative disadvantage of HepG2 cells upon folate deprivation. HepG2 stable cell characterized in **a** were cultured in normal or folate-deprived medium. Direct cell count was performed every 24 h after seeding. Error bars represent cell numbers±s.d. for triplicate experiments. The two-tailed Student's *t*-test was used. **P<0.01; ***P<0.001. (**c**,**d**) K81R and K81Q mutants promote xenograft tumour growth. Subcutaneous xenograft experiment was performed in nude mice using HepG2 stable cells. Major and minor diameters of tumours were measured and tumour volumes were calculated. The two-tailed Student's *t*-test was used. *P<0.05; **P<0.01; ***P<0.001; NS denotes no significance (**c**). 25 days after injection, tumours were dissected, photographed and weighted. The two-tailed Student's *t*-test was used. **P<0.01; NS denotes no significance (**d**). (**e**) The expression of wild-type, K81R and K81Q MAT IIα in xenografts. Whole-cell lysates were prepared from either original HepG2 stable cell lines or xenograft tumours, followed by western blotting analysis. (**f**) The hepatocellular cancer clinical samples show an inverse correlation between MAT IIα protein and K81 acetylation. Human hepatocellular cancer samples each paired with cancerous tissue (designated as C) and adjacent normal tissue (designated as N) were lysed and directly subjected to western blotting. Only eight pairs of samples showcasing inverse correlation are shown. For more samples, please refer to supplementary Fig. 6f. (**g**) Working model. By enhancing MAT IIα association with P300 and UBR4, folate deprivation promotes both MAT IIα K81 acetylation and ubiquitylation, resulting in its proteasomal degradation. Folate stabilizes MAT IIα by dissociating MAT IIα-P300-UBR4 complex, and promoting MAT IIα–HDAC3 interaction. Accumulated MAT II α protein facilitates rapid cell proliferation and, therefore, promotes tumour growth.

Given MAT IIα overexpression was previously implicated to promote cancer cell proliferation and cancer progression^7^,^15^, we next examined the effect of MAT IIα acetylation on cell proliferation and tumour growth. To this end, we generated stable cell lines in HepG2 cells in which endogenous MAT IIα was knocked down by short hairpin RNA (shRNA; targeting 3′-untranslated region of *MAT2A* gene) and the shRNA-resistant MAT IIα wild-type, K81Q or K81R (each contains only coding sequence of wild-type or mutated *MAT2A* gene) was stably expressed. Western blotting analysis demonstrated that the endogenous MAT IIα was effectively knocked down and that wild-type, K81Q or K81R mutants were expressed at levels similar to that of the endogenous MAT IIα (Fig. 6a). Direct cell count and MTT assays were both applied to monitor cell proliferation velocities of wild-type or K81 mutant stable cells under normal or folate-deprived conditions. *MAT2A* knockdown instantly caused growth arrest in HepG2 stable cell line (Supplementary Fig. 6a). HepG2 cells expressing K81R and K81Q mutant stable cells were found proliferating significantly faster than cells expressing wild-type MAT IIα upon folate deprivation, whereas under normal condition, no significant difference in proliferating velocity was detected (Fig. 6b and Supplementary Fig. 6b). As MAT IIα catalyses the production of main methyl donor SAM, and SAM is converted to SAH (S-adenosyl-homocysteine) after the methyltransferation reaction, we then investigated SAM/SAH ratio in different stable cell lines and found that folate deprivation significantly decreased SAM/SAH ratio in wild-type but not in K81R/Q mutant-expressing cells (Supplementary Fig. 6c). As a result, global methylation in wild-type-expressing cells cultured upon folate deprivation was found decreased, compared with that of the mutant-expressing cells grew under the same condition (Supplementary Fig. 6d).

To determine folate's effect on MAT IIα K81 acetylation in tumour growth, we performed xenograft experiments using HepG2 stable cell lines referred previously. Nude mice were subcutaneously injected with HepG2 stable cells and fed with folate-free diet. Tumour growth was continuously monitored thereafter. Measurement of the widths and lengths of tumours demonstrated that HepG2 cells expressing K81R or K81Q mutants displayed a growth rate significantly faster than those expressing wild-type MAT IIα upon folate-deprived dietary (Fig. 6c and Supplementary Fig. 6e). At the time of harvesting, mice injected with cells expressing K81R/K81Q mutants developed larger tumours than those injected with cells expressing wild-type MAT IIα (Fig. 6d). In addition, levels of MAT IIα protein in stable cell lines and xenograft tumours derived from stable cell lines were assessed by western blotting, protein levels of K81R and K81Q mutants were found indeed higher than that of the wild-type MAT IIα (Fig. 6e). Collectively, K81 mutants stabilize MAT IIα and promote tumour cell growth upon folate-deprivation both *in vitro* and *in vivo*.

### K81 acetylation is decreased and inversely correlated with MAT IIα in human hepatocellular cancer

MAT IIα overexpression is implicated in human hepatocellular carcinoma (HCC). The finding that K81Q or K81R mutant promotes HepG2 cell proliferation prompted us to examine K81 acetylation in human hepatocellular cancers. We collected 80 pairs of primary human hepatocellular cancer samples with adjacent normal tissues for direct immunoblotting. Results showed that, compared with the matched normal liver tissues, 40 pairs of samples showed a significant increase of total MAT IIα protein in cancerous tissues (Fig. 6f and Supplementary Fig. 6f). To further exam the correlation between K81 acetylation and MAT IIα protein, we determined MAT IIα K81 acetylation in these 40 pairs of samples, and found that 32 pairs of samples showed relatively higher levels of K81 acetylation in the tumour tissues than that of the matched normal tissues (Fig. 6f and Supplementary Fig. 6g). Furthermore, we found that HDAC3 expression was increased in 19 out 32 HCC samples (Supplementary Fig. 6f). The tumour sample analyses demonstrate that MAT IIα protein levels are elevated in hepatocellular cancers, and K81 acetylation inversely correlates with the elevated MAT IIα protein. These data also indicate an application prospect of using MAT IIα and K81 acetylation as potential biomarkers for hepatocellular cancer diagnosis.

## Discussion

Dysregulation of cellular metabolism is a hallmark of cancer. Besides elevated glycolysis, increased lipogenesis and so on, dysregulation of amino-acid metabolism is also implicated in cancer. Imbalanced methionine metabolism in human liver cancer has been known for at least 60 years. Liver can be considered as the body's SAM factory, and MAT is the key enzyme catalysing biosynthesis of SAM. Overexpression of *MAT2A* is common in various types of cancer^6^,^7^,^15^, whereas knocking down *MAT2A* gene expression causes cell cycle arrest and apoptosis in cancer cells^10^,^11^. Our study reveals a novel mechanism of MAT IIα regulation by acetylation and ubiquitylation in response to folate concentration (Fig. 6g) and provides a new mechanistic insight in MAT IIα upregulation in cancer.

Upregulation of MAT IIα in cancer is in part due to *MAT2A* overexpression regulated by alerted gene transcription or mRNA stability^13^,^14^,^15^. In this study, we report two post-translational modifications of MAT IIα. We propose that MAT IIα acetylation modulated by folate concentration and consequent ubiquitylation play crucial roles in MAT IIα stability control. When folate is sufficient, MAT IIα is stabilized via HDAC3 deacetylation and subsequent inhibition of ubiquitylation. Accumulation of MAT IIα therefore enhances tumour growth. Conversely, when folate is deprived, P300 acetylates MAT IIα at K81. Acetylation therefore promotes UBR4-mediated MAT IIα ubiquitylation, leading to its proteasomal degradation and reduced cell growth.

Folate is needed in large quantities in rapidly proliferating cells. Folate uptake is increased in several types of solid human cancers including ovarian, endometrial, colorectal, kidney, lung and breast carcinomas^20^,^21^,^22^. Anti-folate drugs have long been approved for treating cancers^23^. Therefore, one may expect an important role for folate in MAT IIα regulation.

Indeed, folate-induced MAT IIα stabilization results in an increase in SAM production (Supplementary Fig. 6c). We speculate that SAM accumulation would possibly cause dysregulations of oncogenes and tumour-suppressor genes via affecting global methylation, eventually leading to tumorigenesis and cancer development. In addition, the fact that stabilization of MAT IIα by K81 mutations significantly promotes cancer cell proliferation and *in vivo* tumour growth indicates a critical role of MAT IIα acetylation in coordinating folate availability and the regulation of cell growth and tumorigenesis. It also suggests a possibility that promoting MAT IIα acetylation may merit exploration as a target for cancer therapy. Notably, the observation that MAT IIα is overexpressed in hepatocellular cancer tissues, whereas K81 acetylation is significantly decreased indicates a pivotal role of MAT IIα acetylation regulation in hepatocellular cancer development.

## Methods

### Cell culture and treatment

Cells were all purchased from American Type Culture Collection and cultured in DMEM/high-glucose medium (HyClone) supplemented with 10% fetal bovine serum (HyClone), 1% penicillin and streptomycin at 37 °C, in a humidified atmosphere containing 5% CO_2_. Standard RPMI-1640 and RPMI-1640 without folate were purchased from Life Technologies, Inc. (GIBCO). For folate-deprivation treatment, cells were cultured in the medium containing different amounts of folate as indicated. For TSA, MG132, CHX treatment, please see descriptions in figure legends, respectively.

### Immunoprecipitation and western blotting

Cells were lysed in 0.3% Nonidet P40 buffer (150 mM NaCl, 50 mM Tris-HCl, pH7.5) containing inhibitors (1 mM phenylmethylsulphonyl fluoride, 1 μg ml^-1^ of aprotinin, 1 μg ml^-1^ of leupeptin, 1 μg ml^-1^ of pepstatin, 1 mM Na_3_VO_4_, 1 mM NaF, 30 μM TSA and 15 mM NAM, all in their final concentrations). Cell debris were removed by centrifuging at 4 °C, 13,000 r.p.m. for 15 min, and lysates were incubated for 3 h at 4 °C with anti-flag M2 agarose (Sigma). The immunoprecipitates were washed three times with 0.3% Nonidet P40 buffer before boiled and analysed by western blotting according to the standard methods. The following primary antibodies were commercially obtained: Flag (Sigma, with 1:10,000 working dilution), HA (Santa Cruz, with 1:1,000 working dilution), β-actin (Sigma, with 1:10,000 working dilution), P300 (Santa Cruz, with 1:1,000 working dilution), MAT IIα (Santa Cruz, with 1:1,000 working dilution), acetylated-lysine (Cell Signaling, with 1:1,000 working dilution). Antibody specific to MAT IIα K81Ac was prepared commercially from immunizing rabbits at Shanghai Genomic Inc. (with 1:2,000 working dilution). Images from western blotting have been cropped for presentation. Full-size images are presented in Supplementary Fig. 7.

### Ubiquitin ladder assay

Ubiquitin ladder assay was performed as previously described^24^. Thirty-six hours after transfection, cells were collected and lysed in 1% SDS buffer (50 mM Tris-HCl (pH 7.5), 0.5 mM EDTA, 1 mM dithiothreitol) with inhibitors and boiled for 10 min. Before immunoprecipitation, lysates were diluted ten-fold with 0.3% Nonidet P40 buffer. Ubiquitylation were determined by western blotting.

### siRNA transfection and RNA interference

Downregulation of *UBR4, P300* and *HDAC3* was performed by RNA interference. Synthetic siRNA oligonucleotides were obtained commercially from Shanghai Genepharma Co, Ltd. Sequences of effective sequences are as follow:

si*UBR4-1*: 5′- CGUCCCAGAAUGCCUUAAAdTdT -3′

si*UBR4-2*: 5′- GCUGGUAGUUAUGGUGAAAdTdT -3′

si*UBR4-3*: 5′- GGACCAUCAACCUGUAUUAdTdT -3′

si*P300-1*: 5′- UGACACAGGCAGGCUUGACdTdT -3′

si*P300-2*: 5′- AACAGAGCAGUCCUGGAUUAGdTdT -3′

si*HDAC3*: 5′- CCGCCAGACAAUCUUUGAAdTdT -3′

All siRNA transfections were performed as described in Lipofectamine 2000 (Invitrogen) standard protocol. The knockdown efficiency was verified by western blotting or qPCR.

### Knocking down and putting back stable cell lines

Flag-tagged human wild type, K81R and K81Q mutants of *MAT2A* were cloned into the retroviral vector (pQCXIH) and were co-transfected into HEK293T cells together with vectors expressing *gag* and *vsvg* genes (from vesicular stomatitis virus G). Retroviral supernatants were harvested by filtration (0.45 μm filters) 48 h after initial plasmid transfection and mixed with polybrene (8 μg ml^-1^ as final concentration) to increase the infection efficiency before applied to HepG2 cells. HepG2 cells were infected with the prepared virus for 12 h and screened by hygromycin (350 μg ml^-1^ as final concentration) for at least 2 weeks. pMKO-sh*MAT2A* and pMKO-sh*VEC* were constructed as Short Hairpin RNA vector. shRNA constructs including sh*MAT2A* employed two effective sequences targeting 3′-untranslated region as follows: 5′- GCATAGGTGATCCATGTAACT -3′; 5′- GTAAGTTGGGCTTGCTATTCT -3′. HepG2 stable cells expressing flag-tagged wild type, K81R or K81Q of MAT IIα were infected with pMKO-sh*MAT2A* and pMKO-sh*VEC* retrovirus. MAT IIα knockdown efficiency of positive clones was determined after 2 weeks of drug selection (puromycin, 5 μg ml^-1^ as final concentration).

### GST pull-down assay

BL21 *E. coli* transformed with pGEX**-GST-MAT IIα plasmid was induced (or not induced) by isopropyl-β-D-thiogalactoside (0.1 mM as final concentration) at 20 °C for 12 h. Protein was then purified through GST antibody-conjugated beads. Beads were then added to HEK293T cell lysate (lysed by 0.3% Nonidet P40 buffer), and mixed at 4 °C overnight. Non-induced group was used as a negative control. Beads were subsequently harvested through centrifugation and washed four times by 0.3% Nonidet P40 buffer before boiled by 1 × SDS–polyacrylamide gel electrophoresis loading buffer and subjected to western blotting.

### *In vitro* acetylation assay

Ectopically expressed MAT IIα from BL21 *E. coli* was purified through GST antibody-conjugated beads and incubated with P300 (immunoprecipitation-purified and eluted) in histone acetyl-transferase (HAT) assay buffer (purchased from Millipore), supplemented with acetyl-co-enzyme A (10 μM as final concentration). Reaction was conducted under 30 °C for 1 h with continuous shaking. After adding 5 × SDS–polyacrylamide gel electrophoresis loading buffer and boiled, samples were analysed by western blotting.

### Cell proliferation analysis

5 × 10^4^ HepG2 stable cells were seeded in triplicate in each well of six-well plate, and cultured in normal or folate-deprived medium. Cell numbers were counted every day over a 5-day period via haemacytometer counting method. For MTT (3-(4,5-dimethyl-2-thiazolyl-2,5-diphenyl-2-H-tetrazolium bromide)) assays, 2 × 10^3^ HepG2 stable cells were seeded in quintuplicate in each well of 96-well plate. Standard methods were performed to track the cell proliferation.

### Xenograft analysis

The procedures related to animal studies were approved by the Ethics Committee of the Institutes of Biomedical Sciences, Fudan University. Rodent diet without folate was purchased from Research Diets, Inc. Nude mice (nu/nu, six-week-old males) were fed with folate-deprived diet for 1 week, followed by injected subcutaneously with 5 × 10^6^ HepG2 stable cells. The mice were subsequently fed with folate-deprived diet. Major and minor diameters of tumours were measured every 2 days. Mice were killed after 25 days and tumours were collected and weighed.

### Hepatocarcinoma cancer samples

Hepatocarcinoma cancer samples were obtained from the 10th People's Hospital, Shanghai (Tongji University Affiliated), with written consents from all investigated patients. The procedures related to human subjects were approved by the Ethics Committee of the Institutes of Biomedical Sciences, Fudan University. Direct immunoblotting was performed as mentioned above.

### SAM and SAH quantification

SAM and SAH quantification were performed as previously described^25^. Cells were harvested and weighed. The cell pellets were added with 0.4 M perchloric acid, mixed vigorously and centrifuged. Supernatants were adjusted to pH 5–7 with 2.5 M K_2_HPO_4_ and kept on ice for 15 min to precipitate potassium perchlorate. Samples were centrifuged twice and supernatants were analysed by liquid chromatography-tandem mass spectrometry.

### Methylated DNA quantification

Genomic DNA was prepared using a DNA isolation kit (Beyotime). A methylated DNA quantification kit (Colorimetric; Abnova) was used to detect methylated DNA.

### Statistic analysis

Two-tailed Student's *t*-tests were used for all comparisons, including qPCR analysis. All values included in the figures represent mean±s.d. Error bars represent±s.d. for triplicate experiments. The statistical significance is indicated as asterisks (*). Two-sided *P* value of <0.05 was considered to be statistically significant (*P<0.05, **P<0.01, ***P<0.001).

## Author contributions

H.-B.Y., Y.-Y.X. and X.-N.Z. performed the experiments, analysed the data and co-wrote the manuscript. Y.Z., M.Z. and F.R. contributed to the data analysis. J.-T.L. constructed pGEX-GST-MAT IIα plasmid and contributed data analysis. S.-W.Z. and L.-Y.W. contributed to study of human hepatocarcinoma cancer samples. Y.-Y.X. and S.-W.Z supervised the project. Q.-Y.L. conceived the idea, designed and supervised the study, analysed the data and co-wrote the manuscript.

## Additional information

**How to cite this article:** Yang, H.-B. *et al*. Acetylation of MAT IIα represses tumour cell growth and is decreased in human hepatocellular cancer. *Nat. Commun.* 6:6973 doi: 10.1038/ncomms7973 (2015).

## Supplementary Material

Supplementary Information Supplementary Figures 1-7
