# Supplementary material for: Acetylation of MAT IIα represses tumour cell growth and is decreased in human hepatocellular cancer
Source: Nat Commun. 2015 Apr 30;6:6973. doi: 10.1038/ncomms7973 (PMC4421817; doi:10.1038/ncomms7973)
Supplement: Supplementary Information — Supplementary Figures 1-7 [file ncomms7973-s1.pdf]

a

Position	Gene Names	Mascot Score	PTM Score	Modified Sequence	m/z	Mass Error [ppm]
81	MAT2A;AMS2;MATA2	35.06	137.28	_AAVDYQK(ac)VVR_	595.83	- 2.28

b

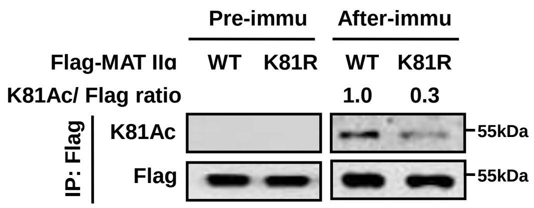

Supplementary Figure 1. MAT IIα is Acetylated at Lysine 81.

(a) Identification of acetylated MAT IIα peptide by mass spectrometry. (**b**) K81R mutant decreases MAT IIα acetylation. Flag-tagged wild type or K81R mutant of MAT IIα was transfected into HEK293T cells and acetylation of the purified proteins was detected using K81Ac antibody or pre-immune serum.

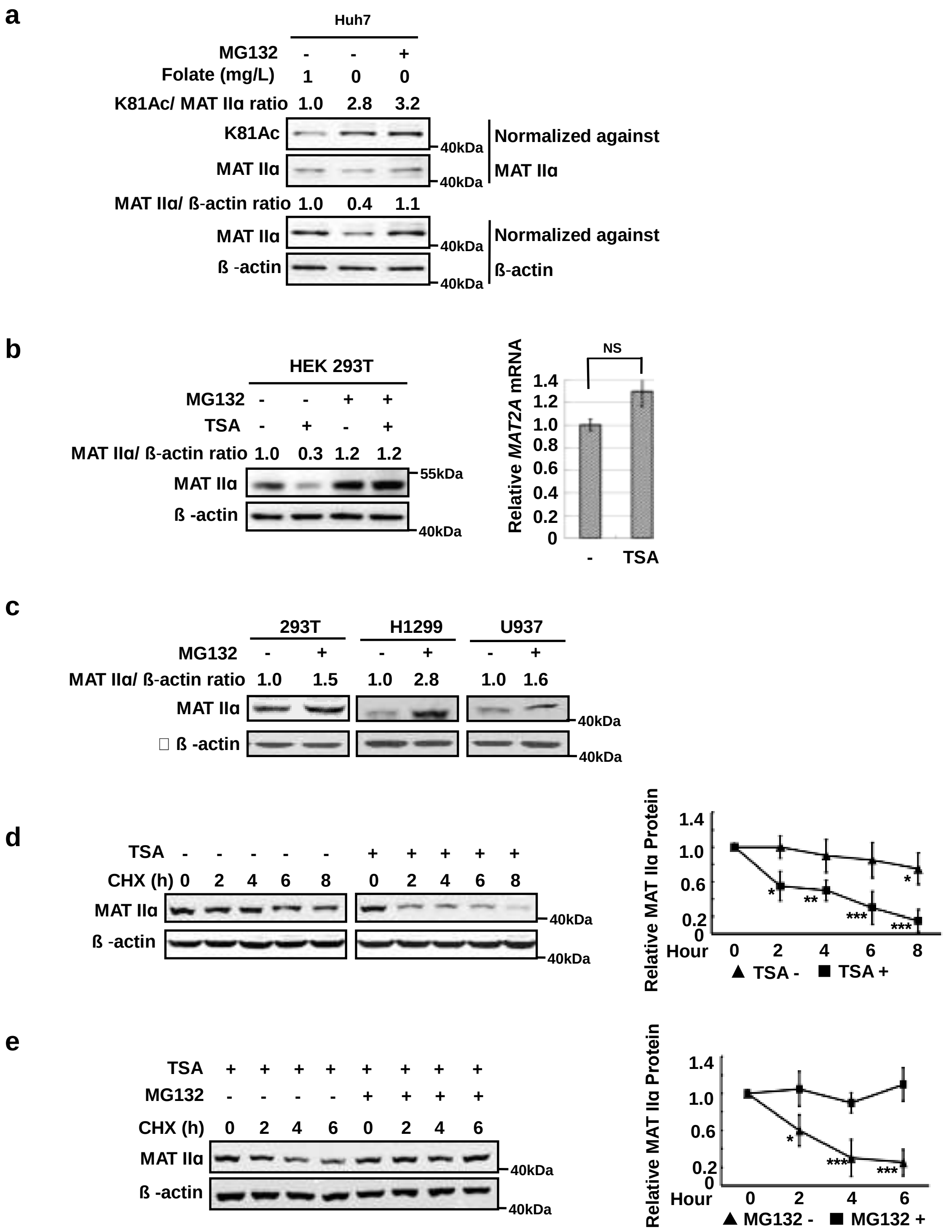

Supplementary Figure 2. Folate-Deprivation Promotes MAT IIα K81 Acetylation and Proteasomal Degradation.

(a) Folate-deprivation increases K81 acetylation of MAT IIα and decreases its protein level. Huh7 cells were cultured upon the indicated condition. Cell lysates were analyzed by western blotting. K81 acetylation levels were normalized against MAT IIα, and MAT IIα protein levels were normalized against β-actin. (**b**) MG132 restores the level of MAT IIα protein reduced by TSA treatment. HEK293T cells were treated

as indicated and cell lysates were analyzed by western blotting. *MAT2A* mRNA was analyzed by qPCR and normalized against *β-actin*. Error bars represent ±SD of triplicate experiments. The two-tailed student *t*-test was used. NS denotes no significance. (**c**) MG132 leads to accumulation of MAT IIα protein. HEK293T, H1299 and U937 cells were treated with either DMSO (solvent) or 10μM MG132. Cell lysates were analyzed by western blotting. (**d**) TSA destabilizes endogenous MAT IIα. HEK293T cells were treated with TSA (10μM for 18h) and CHX (10μg/ml) as indicated. Endogenous MAT IIα protein levels were analyzed by western blotting and normalized against β-actin (left panel). The right panel showcases relative protein amounts of different groups. Error bars represent ±SD of triplicate experiments. The two-tailed student *t*-test was used. * denotes $p < 0.05$; ** denotes $p < 0.01$; *** denotes $p < 0.001$. (**e**) MG132 stabilizes endogenous MAT IIα. HEK293T cells were treated as indicated. MAT IIα protein levels were determined by western blotting and normalized against β-actin (left panel). The right panel showcases relative protein amounts of different groups. Error bars represent ±SD of triplicate experiments. The two-tailed student *t*-test was used. * denotes $p < 0.05$; *** denotes $p < 0.001$.

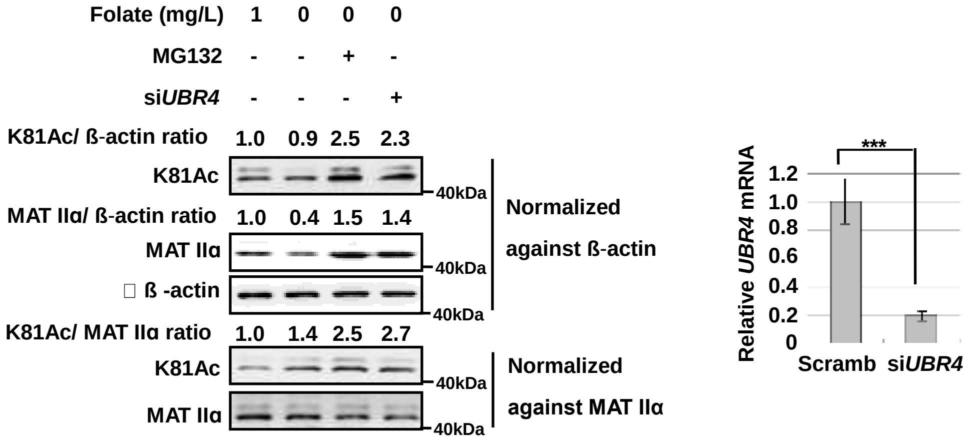

Supplementary Figure 3. UBR4 is the E3 Ligase Mediating MAT IIα Degradation.

UBR4 knockdown increases MAT IIα protein level and its acetylation level. HEK293T cells were transfected with si*UBR4* or control and treated as indicated. MAT IIα protein and its acetylation levels were determined by western blotting (left panel). The efficiency of *UBR4* knockdown was validated by qPCR (right panel). Error bars represent ±SD of triplicate experiments. The two-tailed student *t*-test was used. *** denotes $p < 0.001$.

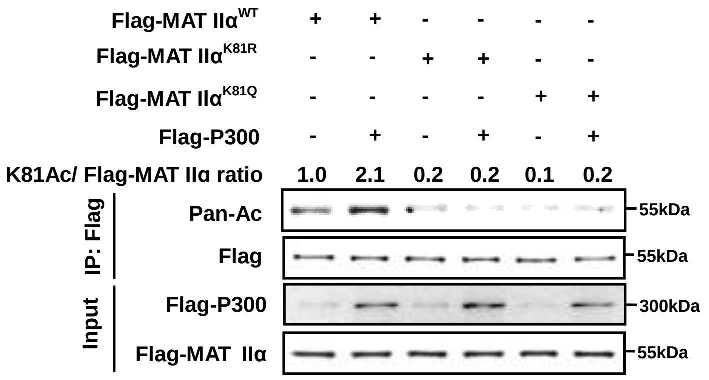

Supplementary Figure 4. P300 Acetylates MAT IIα.

P300 acetylates wild-type MAT IIα but not K81R/Q mutant. HEK293T cells were transfected with indicated plasmids and acetylation of flag-MAT IIα was determined with pan-Acetylation antibody.

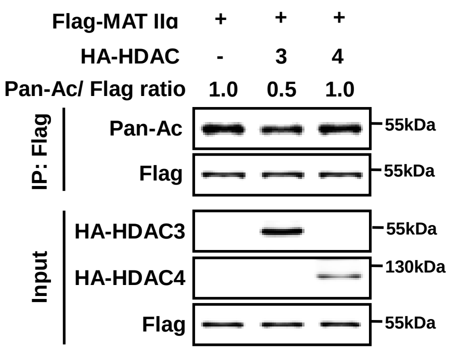

Supplementary Figure 5. HDAC3 Deacetylates MAT IIα

Over-expression of HDAC3, but not HDAC4, decreases the acetylation level of MAT IIα. HA-tagged HDAC3 and 4 were co-transfected respectively with flag-tagged MAT IIα into HEK293T cells and the acetylation levels of MAT IIα were determined by western blotting.

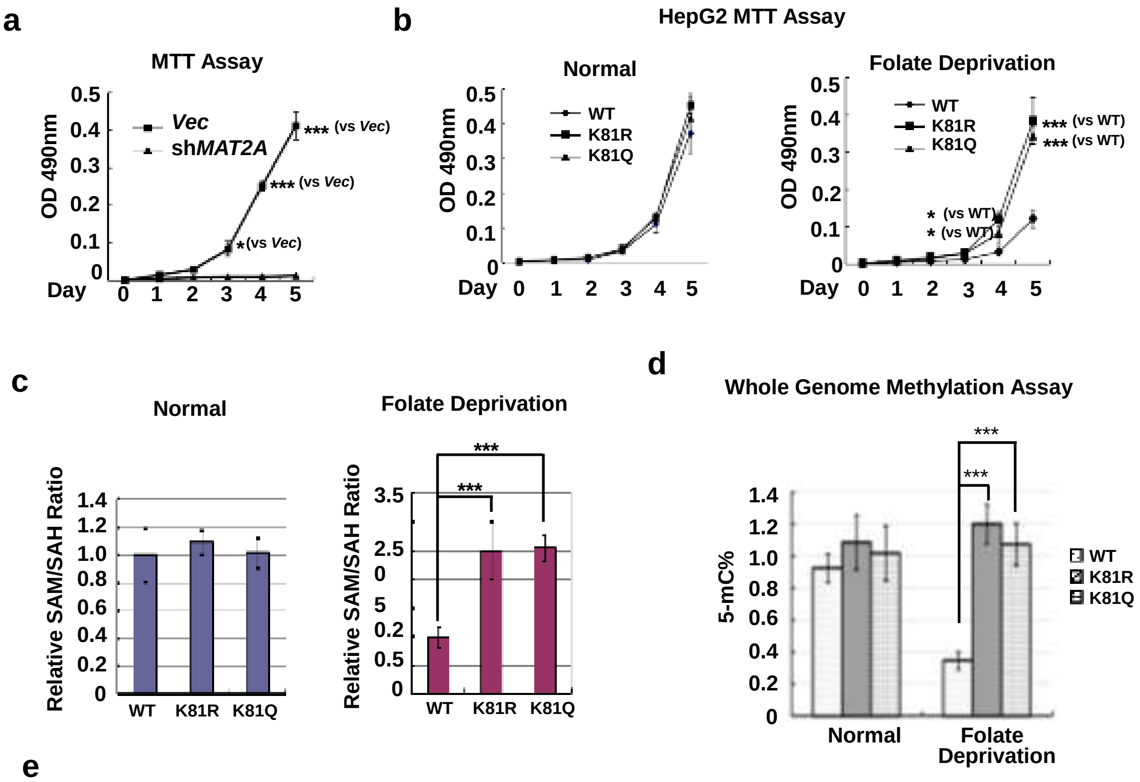

e

Day	12	14	17	19	23	25
p (WT vs K81R)	0.0170	0.0031	0.0011	0.0006	0.0002	0.0004
p (WT vs K81Q)	0.0163	0.0001	0.0001	0.0002	0.0001	0.0001
p (K81R vs K81Q)	0.4850	0.4361	0.9153	0.6437	0.4720	0.5256

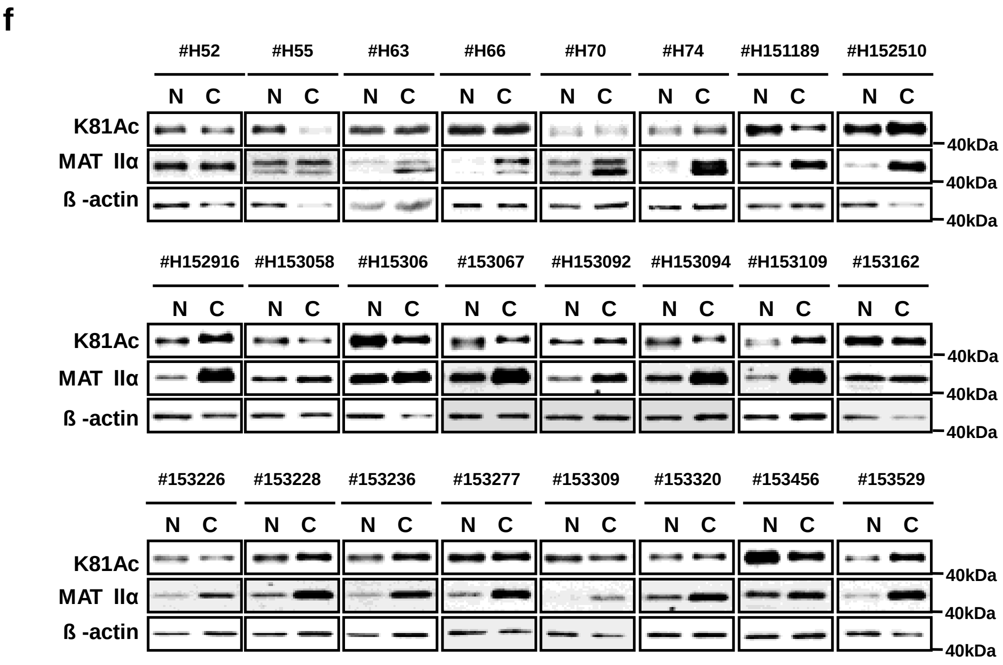

g

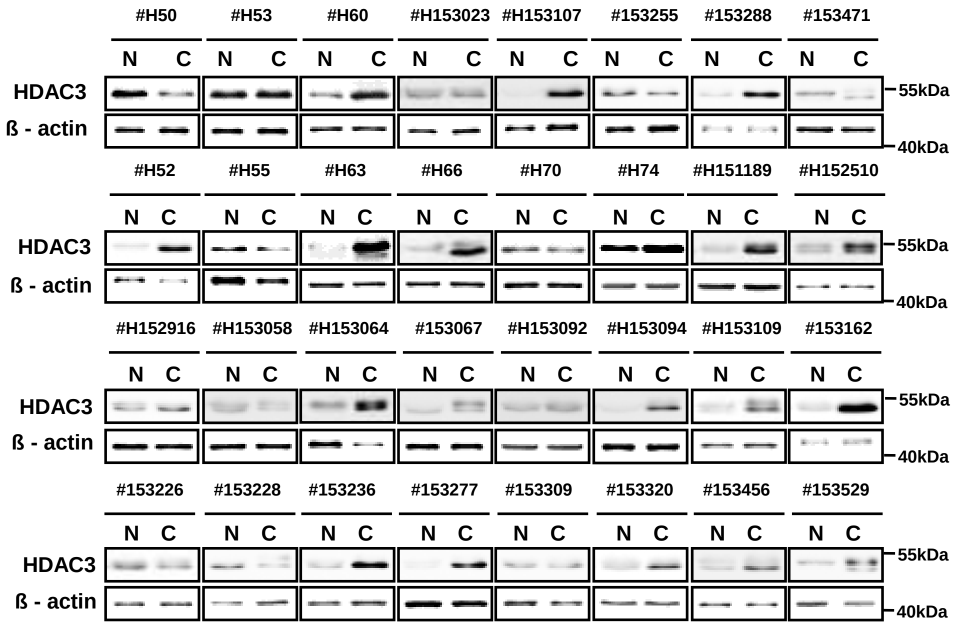

Supplementary Figure 6. K81 Mutation Promotes Tumor Cell Growth *in vitro* and *in vivo*.

(**a**) Knockdown *MAT2A* results in growth arrest in HepG2 stable cells. HepG2 stable cells expressing scramble or sh*MAT2A* were cultured in normal or folate-deprived medium. MTT assays were performed every 24 hours. Error bars represent cell numbers ± SD for triplicate experiments. The two-tailed student *t*-test was used. * denotes $p < 0.05$; *** denotes $p < 0.001$. (**b**) K81R and K81Q mutations reverse the proliferative disadvantage of HepG2 cells upon folate-deprivation. HepG2 stable cells were cultured in normal or folate-deprived medium. MTT assays were performed every 24 hours. Error bars represent cell numbers ± SD for triplicate experiments. The two-tailed student *t*-test was used. * denotes $p < 0.05$; *** denotes $p < 0.001$. (**c**) Folate-deprivation reduces SAM/SAH ratio in wild-type MAT IIα-expressing stable cells. HepG2 stable cells were cultured with or without folate for 48h before harvest and weighed. The cell pellets were added with 0.4 M perchloric acid (100 μL per 30 mg pellet), mixed vigorously and centrifuged. pH of supernatants were adjusted to 5-7 with 2.5 M K2HPO4 and kept on ice for 15 min allowing potassium perchlorate to precipitate. Samples were centrifuged twice and supernatants were analyzed by LC−MS/MS. The statistical significance of difference among different groups was evaluated using two-tailed student *t*-test. *** denotes $p < 0.001$. (**d**) Folate-deprivation decreases genomic DNA methylation of HepG2 stable cells expressing wild-type MAT IIα but not K81 mutants. HepG2 stable cells were cultured with or without folate for 48h before harvest. Genomic DNA was isolated, and Methylated DNA quantification kit (Abnova co.) was used to detect total DNA methylation. The statistical significance of difference among different groups was evaluated using two-tailed studen *t*-test. *** denotes $p < 0.001$. (**e**) *p* values in figure 6c were shown. (f) The hepatocellular cancer clinical samples show an inverse correlation between MAT IIα protein and K81 acetylation. Human hepatocellular cancer samples each paired with cancerous tissue (designated as C) and adjacent normal tissue (designated as N) were lysed and directly subjected to western blotting. (g) HDAC3 expression is increased in 19 out 32 (about 59%) cases of HCC samples. For technical details, please refer to Figure legend of Fig. 6 in the main figures.

Supplementary Figure 7. Full Scans of Western Blotting Data in Main Figures.

Figure 1a

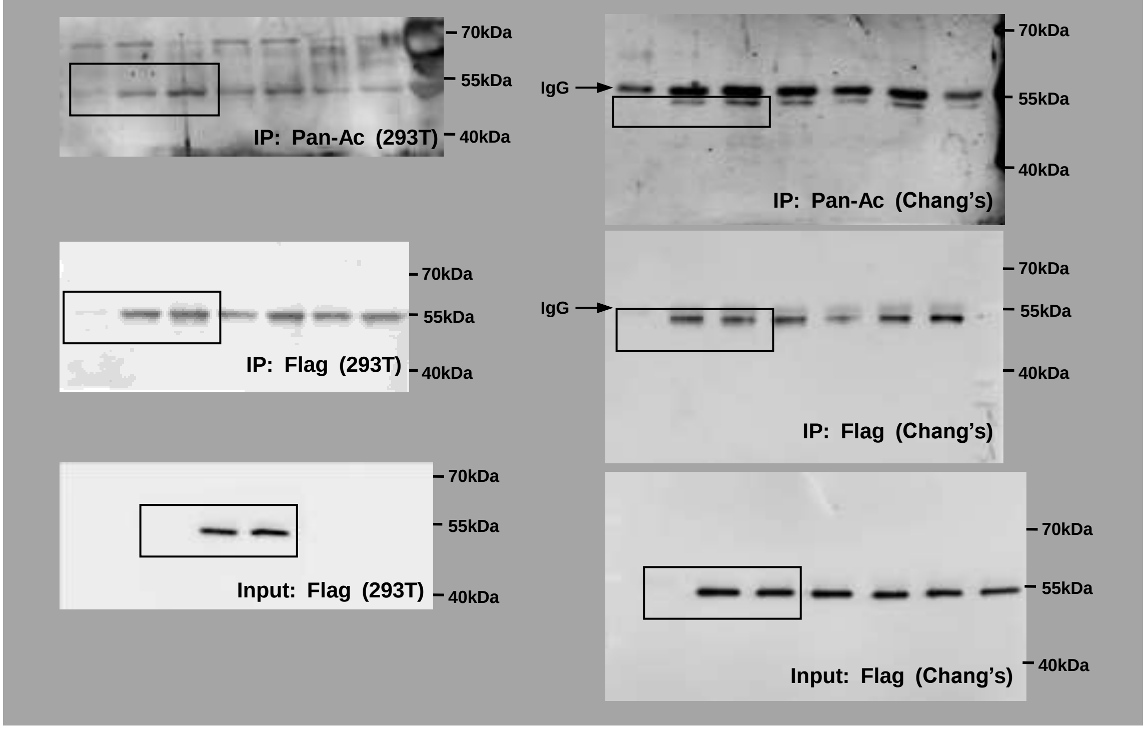

Figure 1c

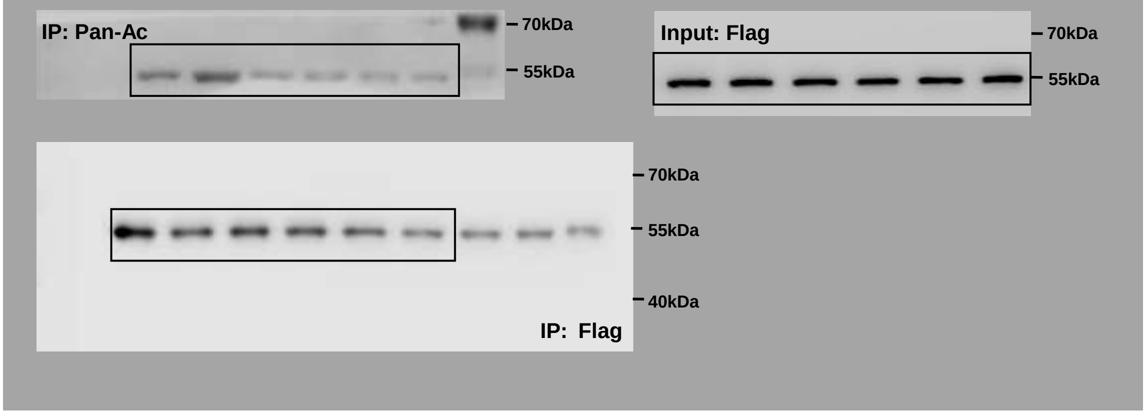

Figure 1e

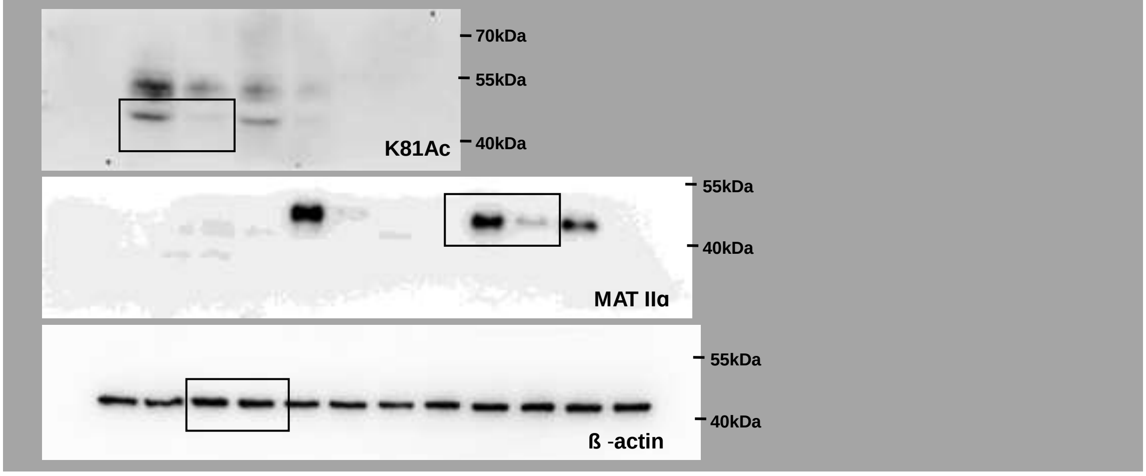

Figure 1f

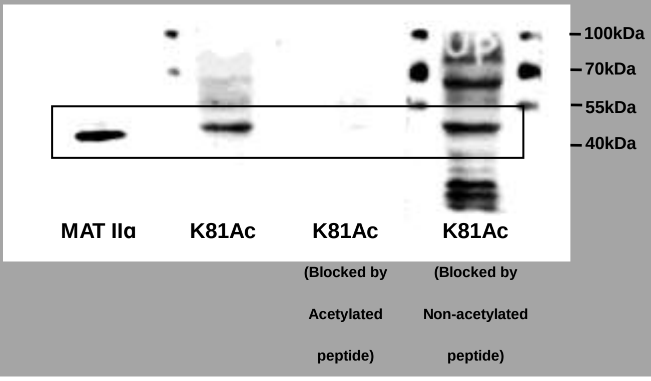

Figure 1g

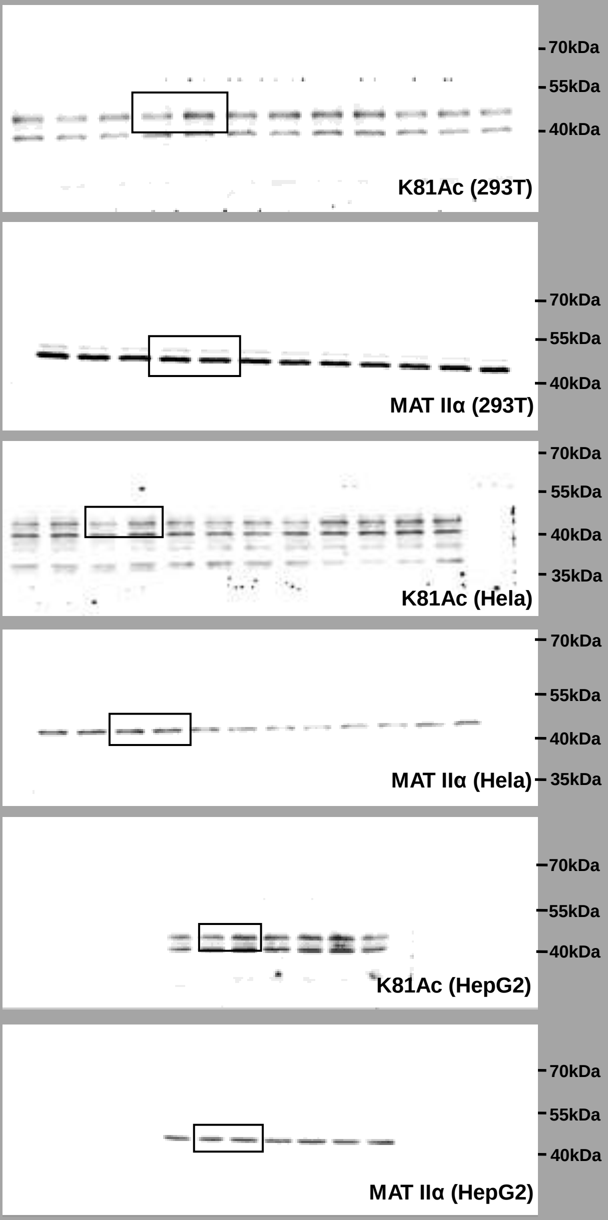

Figure 2a

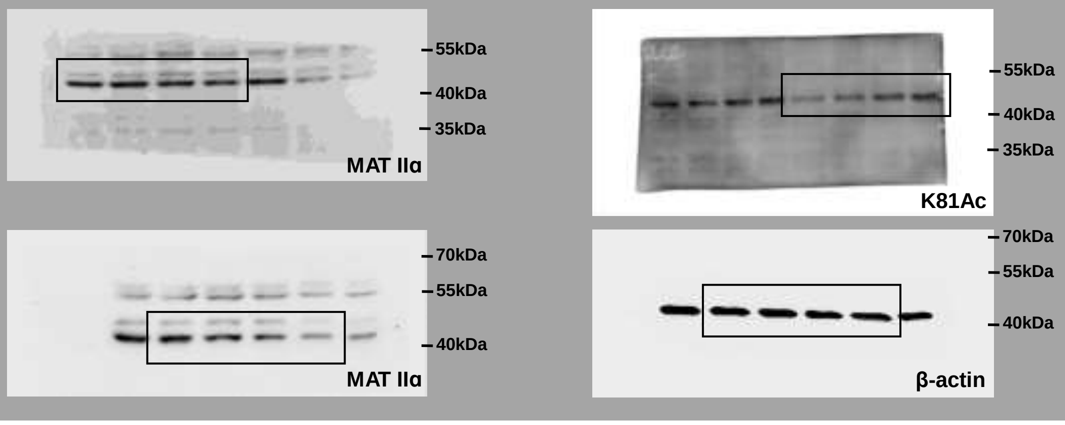

Figure 2d

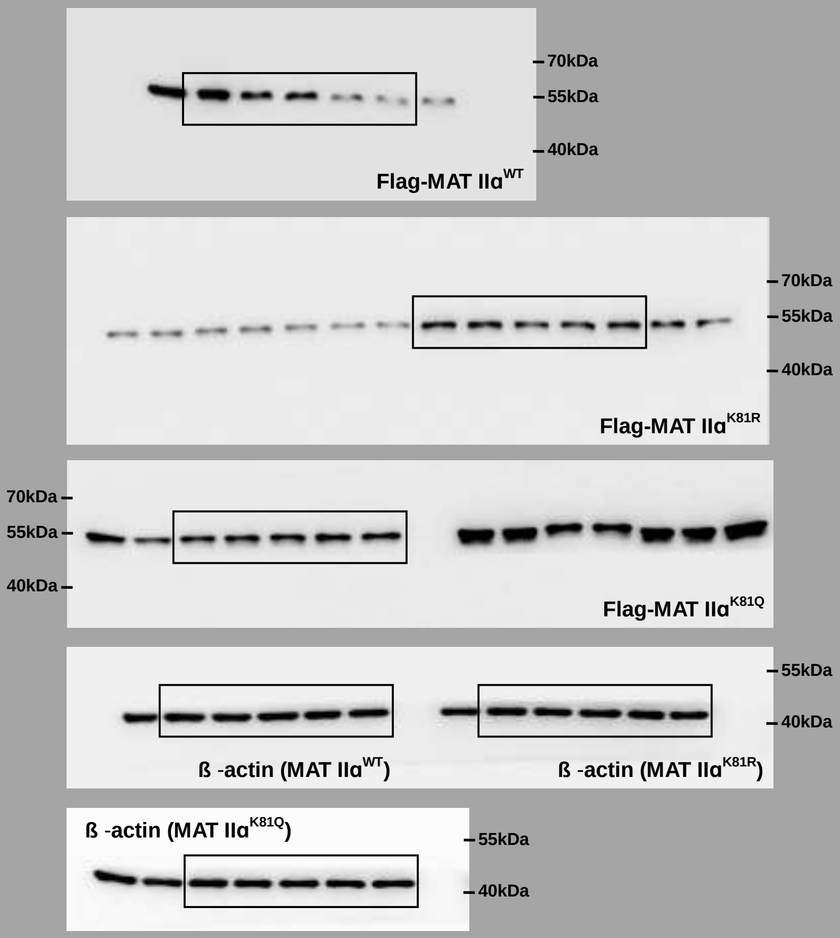

Figure 2g

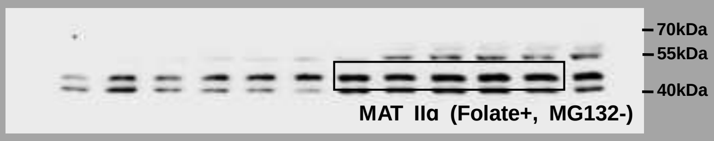

Figure 2g

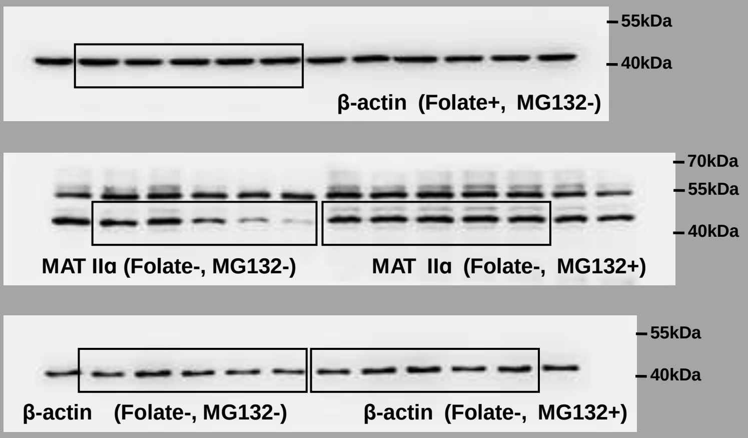

Figure 3a

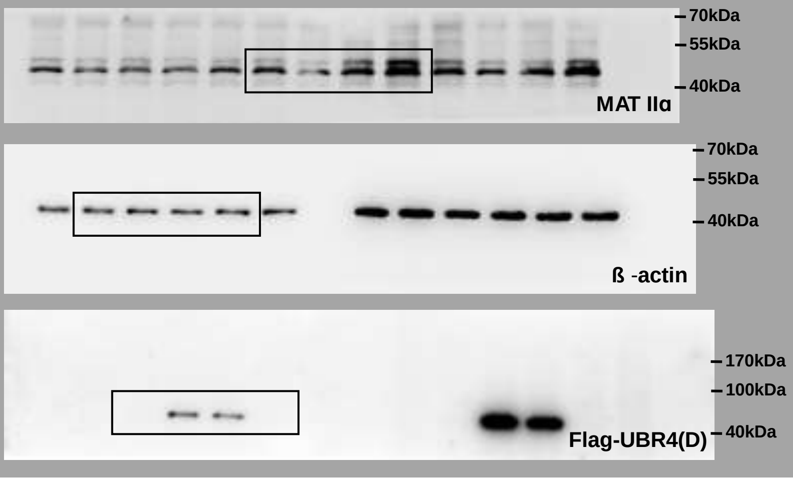

Figure 3b

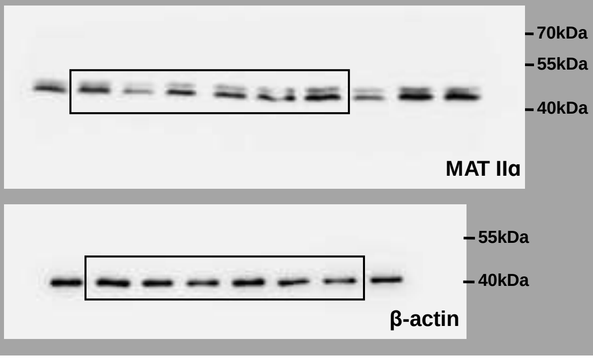

Figure 3c

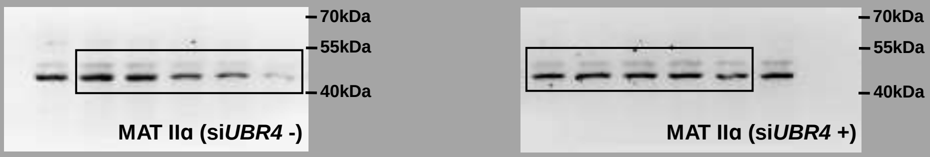

Figure 3c

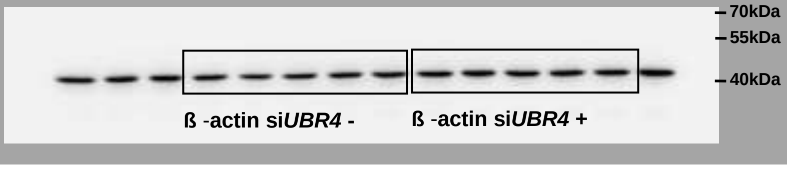

Figure 4a

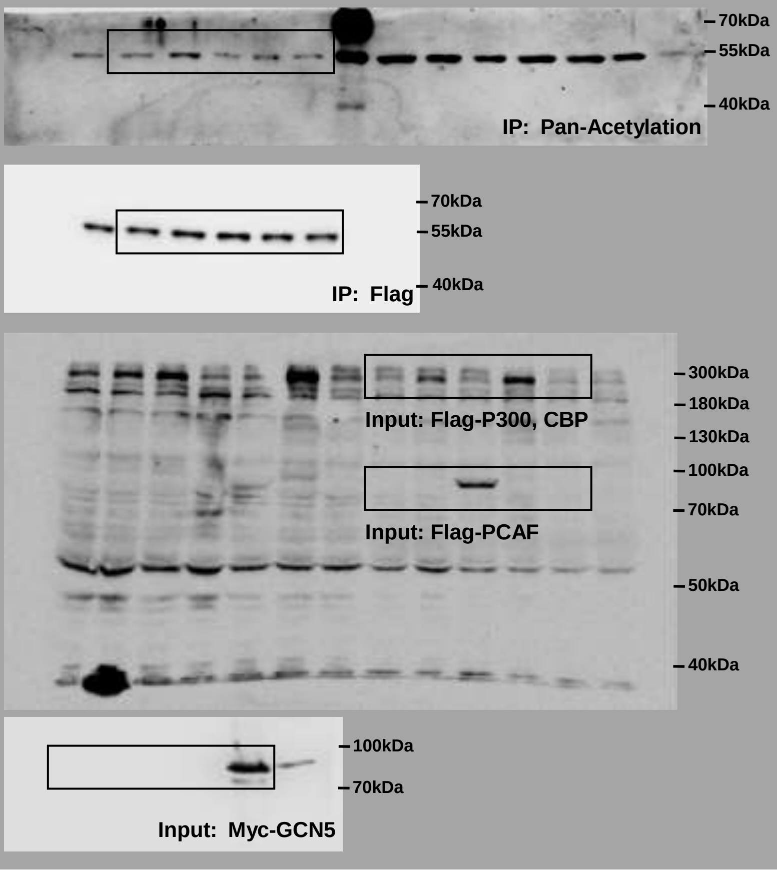

Figure 4b

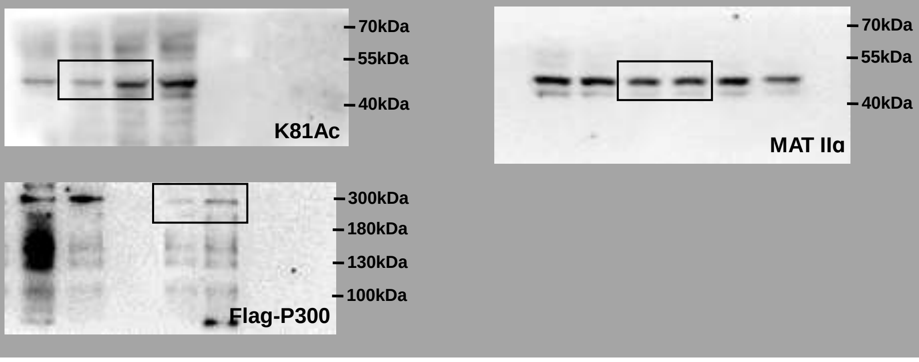

Figure 4c

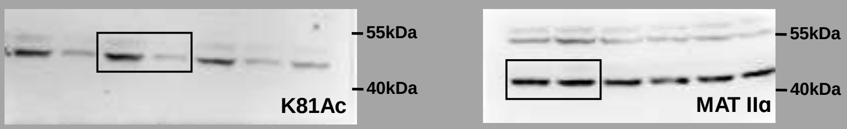

Figure 4c

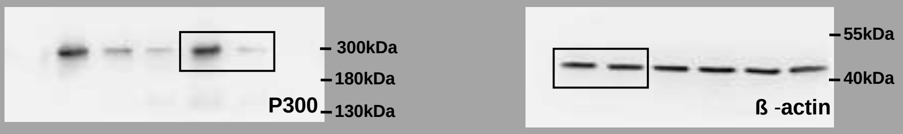

Figure 4d

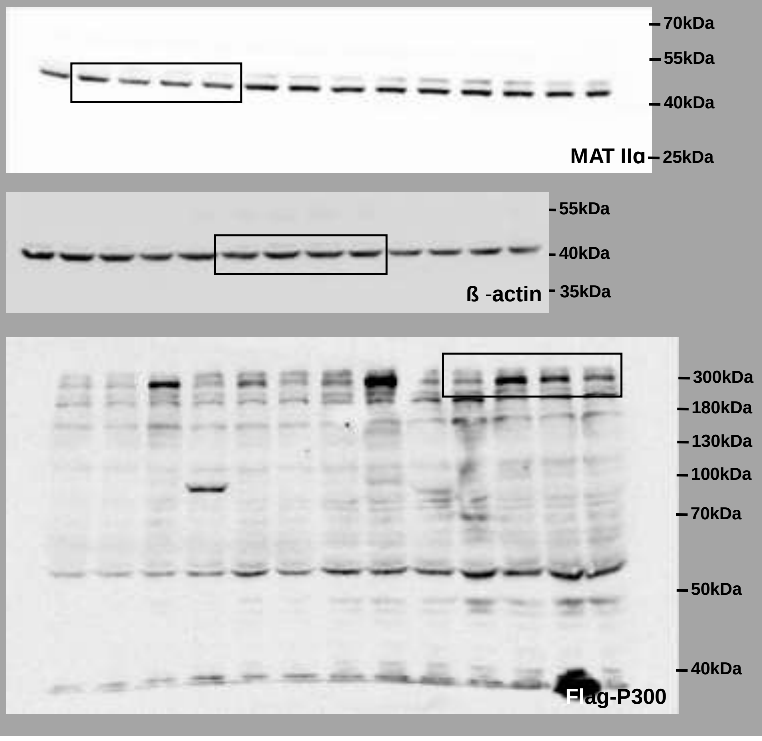

Figure 4e

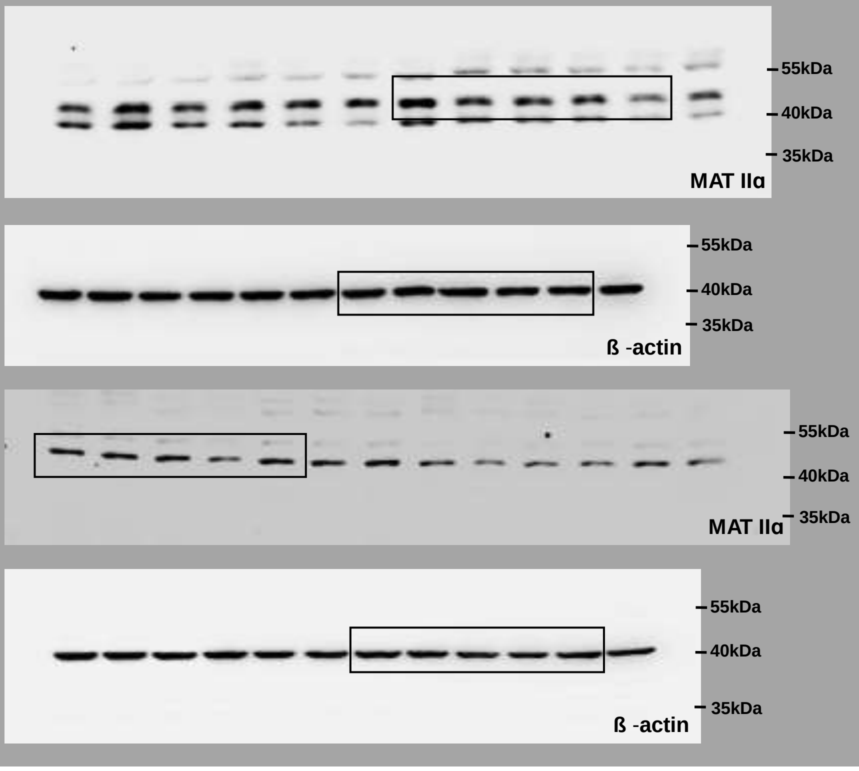

Figure 4f

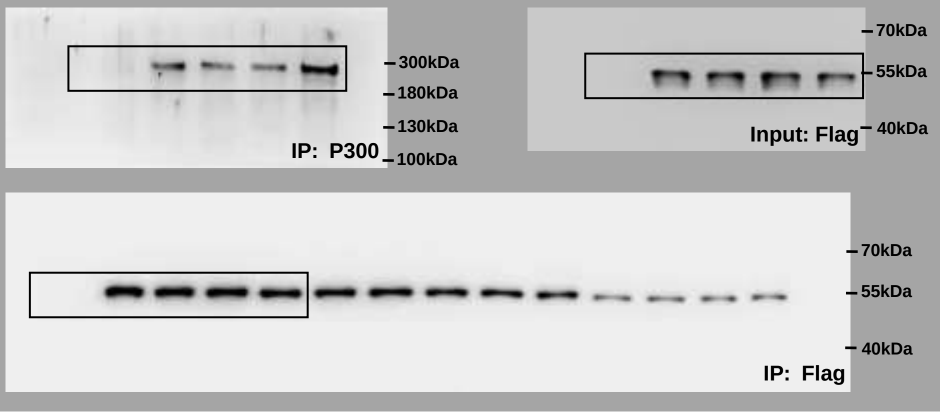

Figure 4g

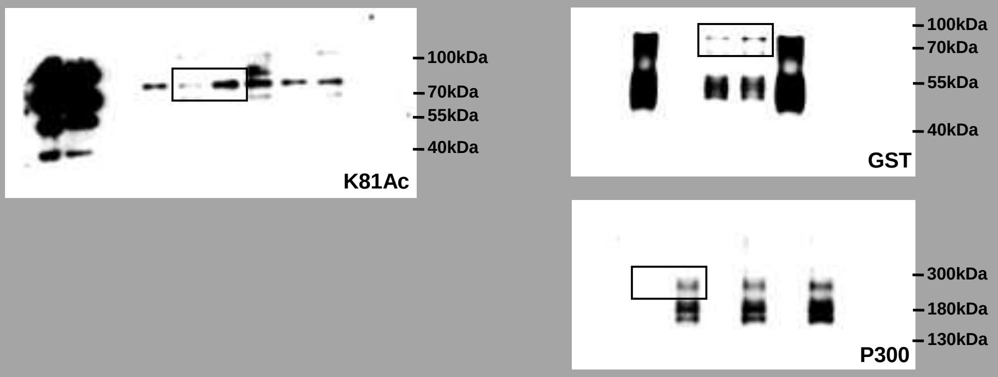

Figure 5a

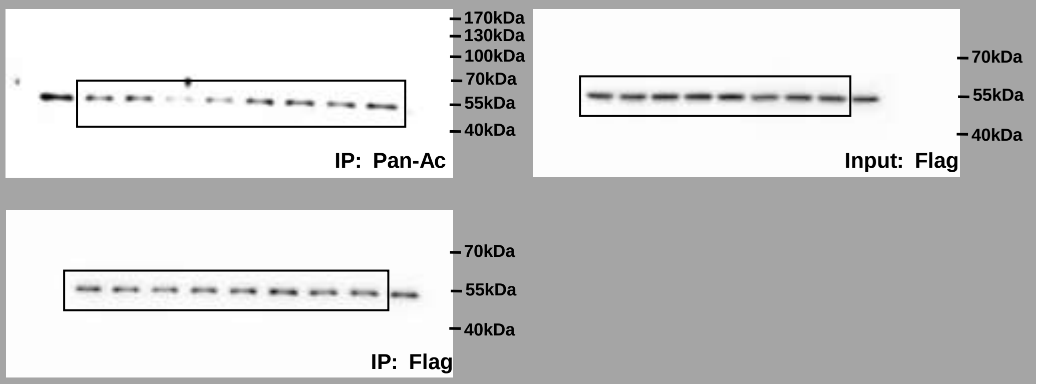

Figure 5b

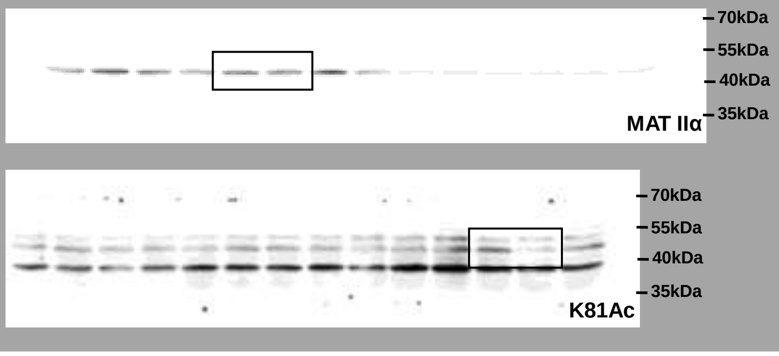

Figure 5c

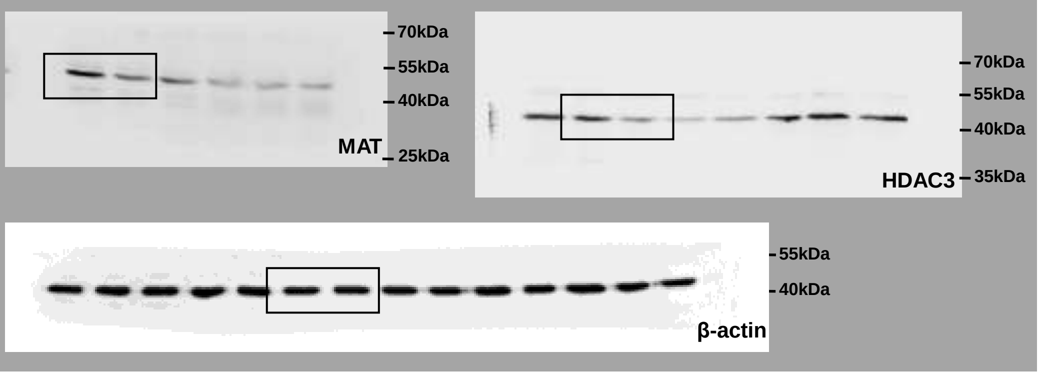

Figure 5d

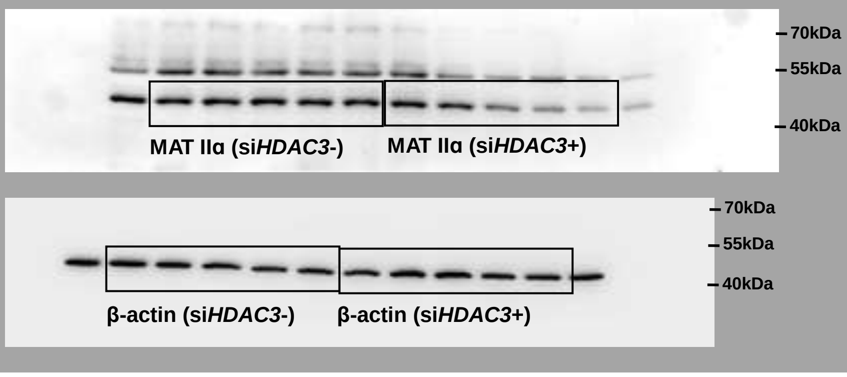

Figure 5e

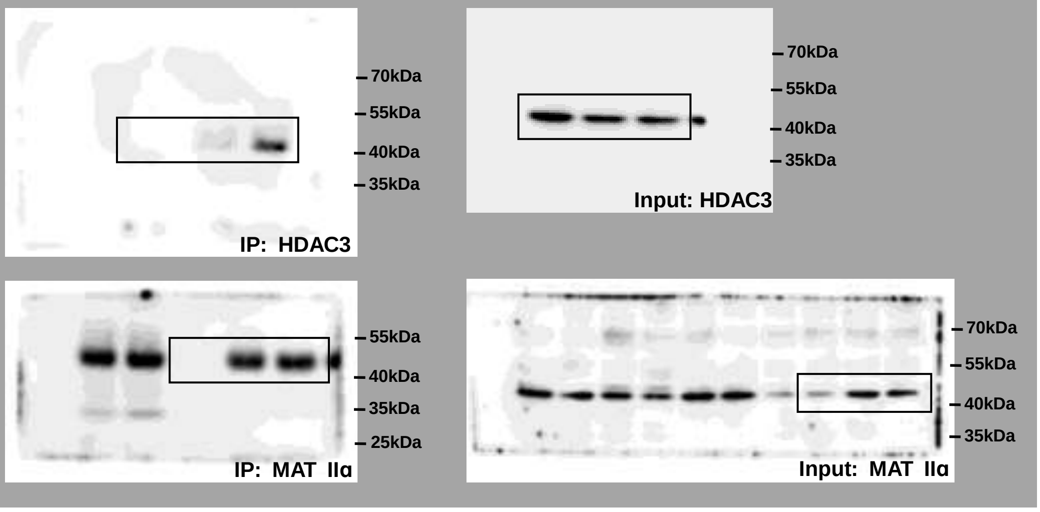

Figure 5f

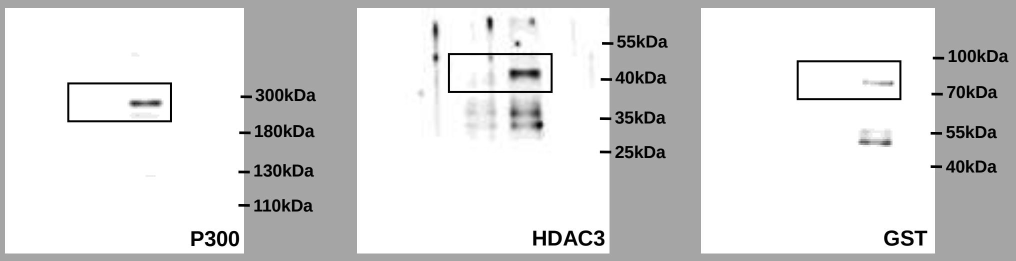

Figure 6a

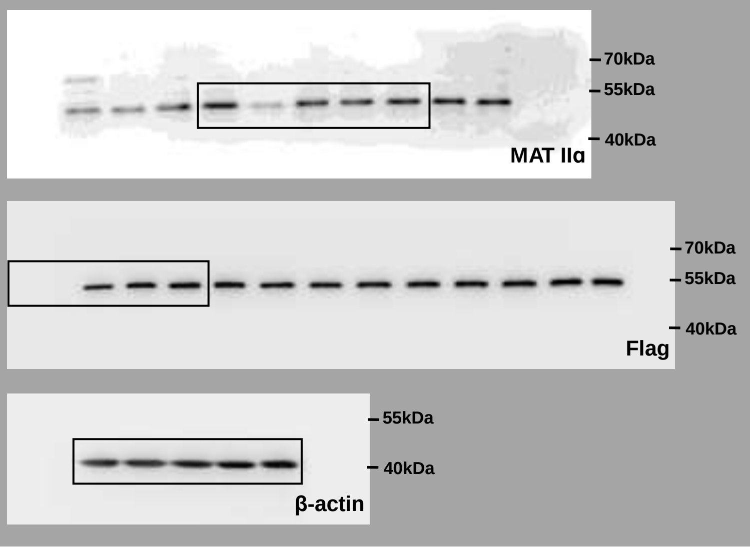

Figure 6e

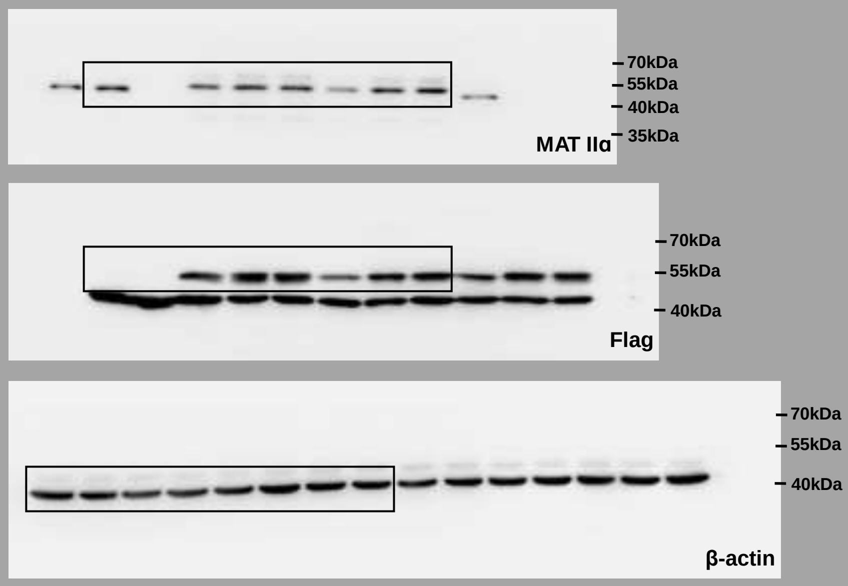

Figure 6f

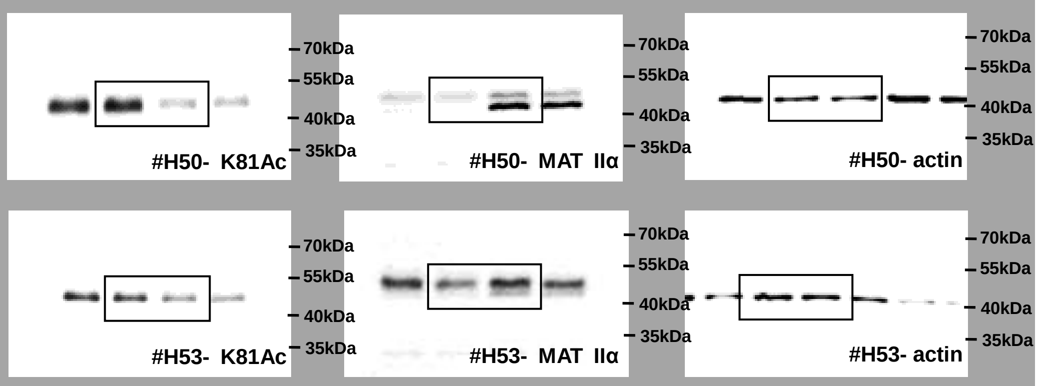

Figure 6f

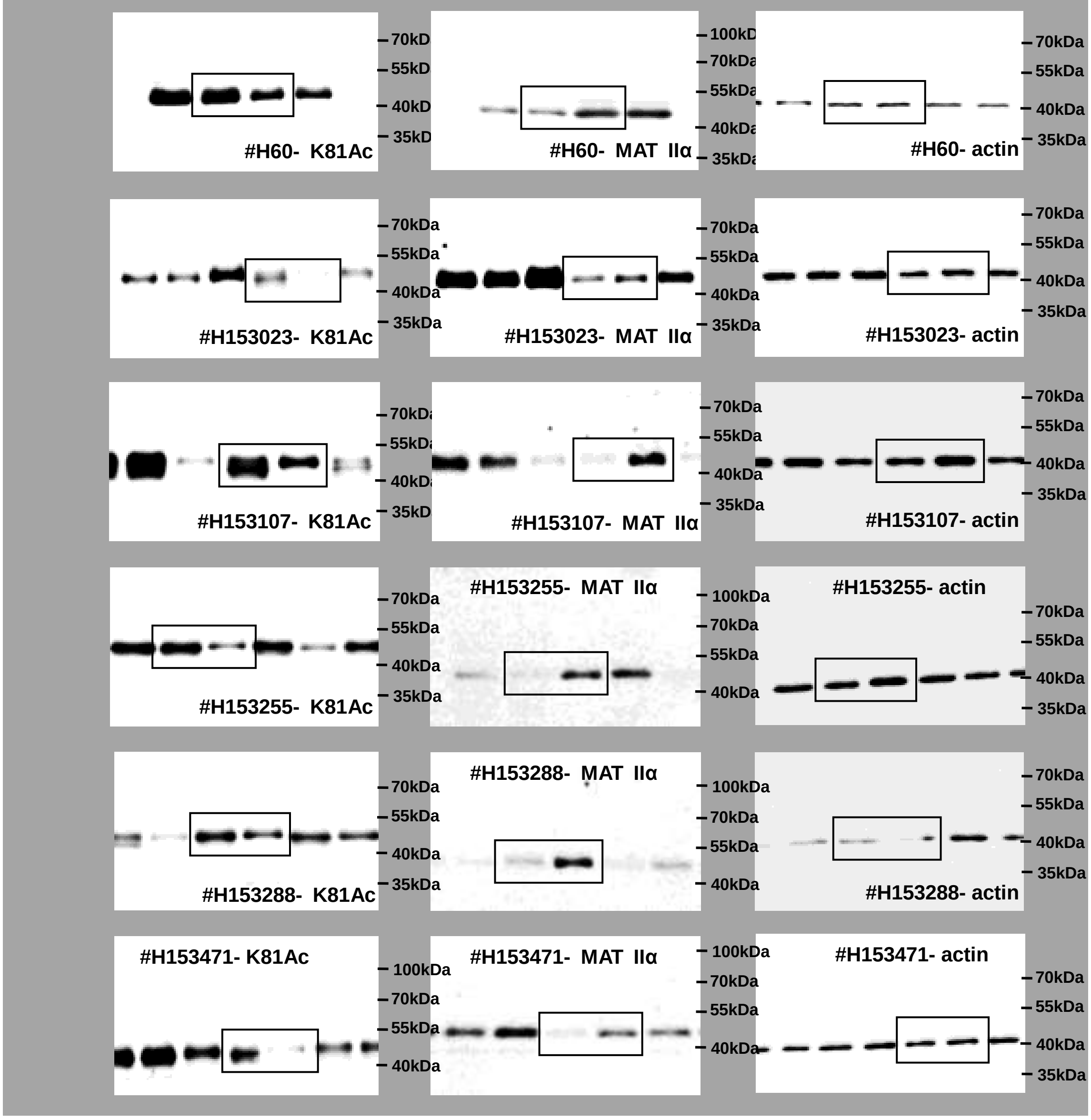

#H60- K81Ac
70kD
55kD
40kD
35kD
#H60- MAT IIα
100kD
70kDa
55kDa
40kDa
35kDa
#H60- actin
70kDa
55kDa
40kDa
35kDa
#H153023- K81Ac
#H153023- MAT IIα
#H153023- actin
#H153107- K81Ac
#H153107- MAT IIα
#H153107- actin
#H153255- K81Ac
#H153255- MAT IIα
#H153255- actin
#H153288- K81Ac
#H153288- MAT IIα
#H153288- actin
#H153471- K81Ac
#H153471- MAT IIα
#H153471- actin
100kDa
70kDa
55kDa
40kDa
35kDa
